# Red blood cell-conjugated biomimetic nanomedicine for enhanced therapy of non-small cell lung cancer

**DOI:** 10.7150/thno.121646

**Published:** 2026-01-21

**Authors:** Seok Theng Chiang, Yueping Jin, Qian Zhao, Hongju Ling, Qinghua Xia, Tianzhen Han, Rongxiu Li, Weidong Li, Zhaohui Lan, Xiangzhao Ai, Haijiao Lu

**Affiliations:** 1 Department of Respiratory and Critical Care Medicine, Shanghai Chest Hospital, Shanghai Key Laboratory of Thoracic Tumor Biotherapy, Shanghai Jiao Tong University, School of Medicine, Shanghai, 200030, China.; 2 Department of Bioengineering, School of Life Sciences and Biotechnology, Shanghai Jiao Tong University, Shanghai 200240, China.; 3 Shanghai Lung Cancer Center, Shanghai Chest Hospital, Shanghai Jiao Tong University, School of Medicine, Shanghai 200030, China.; 4 Center for Brain Health and Brain Technology, Global Institute of Future Technology, Shanghai Jiao Tong University, Shanghai 200240, China.; 5 Urology Department, Shandong Provincial Hospital, Jinan, 250021, China.

**Keywords:** biomimetic nanoparticle, red blood cell, drug delivery, non-small cell lung cancer, osimertinib

## Abstract

**Rationale:** Epidermal growth factor receptor tyrosine kinase inhibitors (EGFR-TKIs) are a standard therapy for non-small cell lung cancer (NSCLC). Despite their clinical efficacy, dose-limiting systemic toxicity and the eventual development of acquired resistance limit their long-term benefit. Therefore, innovative drug delivery strategies are highly demanded to optimize the therapeutic window, minimizing toxicity of EGFR-TKIs at lower doses without compromising their efficacy.

**Methods:** We developed a red blood cell (RBC)-based biomimetic platform for the systemic delivery of EGFR-TKIs. Osimertinib-loaded poly(lactic-co-glycolic acid) nanoparticles were camouflaged with a biotinylated RBC membrane (Osi-RNPs). They were then conjugated to the surface of RBCs *via* high-affinity biotin-streptavidin interactions to form a stable construct (Osi-RNP-SA-RBC). The physicochemical characteristics, cellular uptake, and *in vitro* antitumor activity of Osi-RNPs were characterized. We further assessed the pharmacokinetics, biodistribution, therapeutic efficacy, and safety profile of Osi-RNP-SA-RBC in subcutaneous and orthotopic NSCLC mouse models.

**Results:** The Osi-RNP-SA-RBC platform demonstrated stable attachment, favorable hematocompatibility and excellent biosafety. Compared to the Osi-RNP-RBC (nonspecific adsorption), Osi-RNP-SA-RBC presented prolonged blood circulation (1.6-fold) and enhanced tumor accumulation (2.2-fold). Upon intravenous injection of Osi-RNP-SA-RBC at a reduced dose and frequency, superior tumor suppression was observed in both subcutaneous (16.8-fold increase) and orthotopic (4.2-fold increase) NSCLC mouse models compared to the free osimertinib at same administration dosage.

**Conclusion:** This study demonstrates the potential of RBC-conjugated biomimetic nanomedicine as a promising strategy for enhancing the treatment efficiency of EGFR-TKI against NSCLC *in vivo*.

## Introduction

Lung cancer remains the most commonly diagnosed malignancy and the leading cause of cancer-related mortality worldwide. It accounts for approximately 2.5 million new cases and an estimated 1.8 million deaths in 2022 1. Non-small cell lung cancer (NSCLC) is the most prevalent type (~85%) of lung cancer, with the majority being diagnosed at a locally advanced or metastatic stage. Generally, surgery and chemotherapy remain the standard treatment for NSCLC patients. The discovery of oncogenic driver mutations, including the alterations in epidermal growth factor receptor (EGFR) gene and rearrangement of the anaplastic lymphoma kinase (ALK) gene, has completely changed the therapeutic landscape from platinum-based chemotherapy to molecular targeted therapy for NSCLC patients 2. EGFR is a transmembrane tyrosine kinase receptor that widely regulates various signaling pathways involved in cell proliferation, migration, and survival. The overexpression of EGFR is frequently detected in NSCLC, with a prevalence of 19% to 89%, and is associated with poor prognosis. With advances in precision medicines, EGFR tyrosine kinase inhibitors (TKIs) have shown exceptional efficacy in targeting EGFR signaling and inducing antitumor responses that have improved patient outcomes and quality of life. Despite the initially pronounced responses to EGFR-TKIs such as the first-generation inhibitors (*e.g.*, erlotinib, gefitinib, and icotinib) and second-generation inhibitors (*e.g.*, afatinib and dacomitinib), their therapeutic efficacy is often compromised in NSCLC patients by dose-related systemic toxicity, while the subsequent emergence of acquired resistance with secondary mutation further limits long-term therapeutic efficacy, typically within 9 to 14 months of treatment. Osimertinib, the third-generation TKI, was specifically designed and subsequently approved for the treatment of patients harboring EGFR-sensitizing or T790M-mediated resistance mutations. It exhibits superior efficacy in EGFR-mutated NSCLC patients, with a median overall survival of 38.6 months 3, 4. However, even with improved selectivity, its long-term administration is frequently constrained by cumulative, dose-limiting toxicities that prevent dose escalation and limit treatment duration. Concurrently, the inevitable emergence of acquired resistance leads to therapeutic failure and tumor progression 5. To address these limitations, combination therapies incorporating conventional chemotherapeutics or immune checkpoint inhibitors are increasingly explored to enhance therapeutic outcomes. However, tumor cells can adapt through diverse mechanisms to evade treatment, leading to the development of multidrug resistance and diminished therapeutic efficacy over time 6. Consequently, therapeutic options become progressively limited, presenting a significant clinical challenge in the management of advanced, TKIs-resistant NSCLC patients.

In clinical settings, EGFR-TKIs are typically administered once or twice daily at their maximum tolerated dose due to their relatively short half-life in the blood. The high dosages of TKIs have been reported to induce off-target toxicities in different organs such as the heart, liver, kidneys, eyes, and skin, and can contribute to the development of drug resistance 7, 8. Consequently, a major therapeutic challenge is optimizing the dosing regimen to minimize TKIs-related toxicity without compromising efficacy, given the narrow therapeutic window that is difficult to achieve with conventional administration 9. Nanomedicines have emerged as a promising strategy to address this challenge, aiming to enhance therapeutic efficacy by improving drug delivery precision while reducing systemic side effects 10-19. However, many nanocarriers exhibit insufficient blood circulation time following intravenous (*i.v.*) administration. Polyethylene glycol (PEG) conjugation is a common approach to shield nanoparticles (NPs) from plasma proteins adsorption, thereby reducing clearance by the reticuloendothelial system (RES) and improving delivery efficacy 20. A significant limitation of this strategy is the accelerated blood clearance (ABC) phenomenon, whereby PEGylated NPs are rapidly removed upon repeated administration due to immune recognition and anti-PEG antibody production, despite acceptable circulation after a single dose 21. Given the long circulatory lifespan (~120 days in humans) and inherent biocompatibility of red blood cells (RBCs), Zhang's group first developed a biomimetic nanoplatform by cloaking RBC membranes onto the surface of biodegradable poly(lactic-co-glycolic acid) (PLGA) NPs, which exhibited a prolonged circulation compared to PEGylated liposomes 22. Subsequent studies have confirmed that RBC-camouflaged NPs offer advantages in pharmacokinetics and therapeutic efficacy 23-32. Nevertheless, the RBC-mimicking NPs still face limitations, including a suboptimal circulatory half-life attributable to their nanoscale morphology and potential membrane damage during fabrication 33, 34. An alternative strategy, termed RBC hitchhiking, involves the nonspecific adsorption of NPs onto RBC surface *via* hydrophobic, van der Waals, or electrostatic interactions 35-37. This approach leverages pulmonary capillary shear forces to dislodge the carriers, showing its applicability in lung-targeted drug delivery 38-49. However, for systemic delivery, the instability of this adsorption results in rapid NP detachment in circulation, which limits its utility compared to the enduring carriage capacity of native RBCs. Therefore, a stable and efficient biomimetic delivery platform is highly demanded to minimize toxicity of EGFR-TKIs at lower doses while maintaining their therapeutic effectiveness.

**Figure 1:**
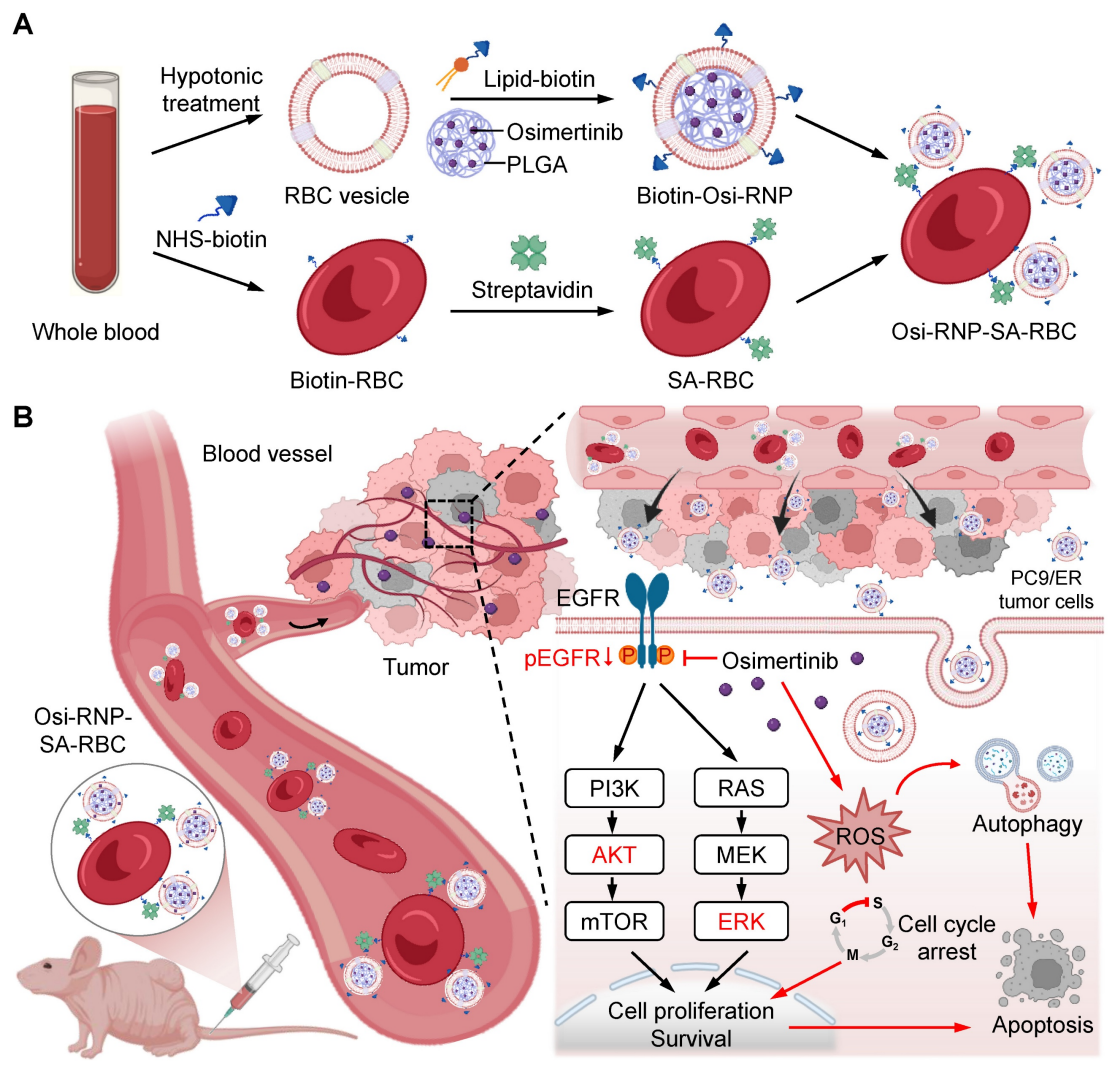
** Preparation and mechanism of RBC-conjugated biomimetic nanocarriers.** (**A**) Schematic illustration of nanocarrier fabrication. Osimertinib-loaded PLGA NPs were encapsulated within biotinylated RBC membrane to generate biomimetic Osi-RNPs. Subsequently, the Osi-RNPs were conjugated to streptavidin (SA)-modified RBCs *via* high-affinity biotin-SA binding, yielding stable Osi-RNP-SA-RBC constructs for systemic administration. (**B**) Proposed therapeutic mechanism *in vivo*. Following intravenous injection, the Osi-RNP-SA-RBC platform prolongs circulation time and minimizes premature clearance, enhancing tumor accumulation and drug bioavailability. At the tumor site, Osi-RNPs are internalized *via* endocytosis, leading to intracellular release of osimertinib. The released drug inhibits the PI3K/AKT and MAPK/ERK signaling pathways, induces cell cycle arrest, elevates ROS levels, and activates autophagy. These coordinated effects promote tumor cell apoptosis while minimizing off-target toxicity.

Inspired by the inherent biocompatibility and prolonged circulatory lifespan of RBCs, we hypothesize that the stable conjugation of biomimetic nanomedicine onto the RBCs surface, as opposed to non-specific adsorption, could significantly enhance the pharmacokinetic profile of EGFR-TKIs. This improvement was anticipated to augment therapeutic efficacy against malignant tumors. Herein, we developed a biomimetic delivery system in which osimertinib was encapsulated within biotinylated RBC membrane-camouflaged NPs (denoted “Osi-biotin-RNP”). These biomimetic NPs are then conjugated onto biotinylated RBCs *via* high-affinity biotin-streptavidin (SA) interaction, forming a stable construct (denoted “Osi-RNP-SA-RBC”) (**Figure 1**). Our results indicate that the Osi-RNP-SA-RBC platform facilitates stable and secure attachment of EGFR-TKI-loaded NPs to RBCs, leading to prolonged circulation time and enhanced tumor accumulation. Consequently, this system significantly potentiated the therapeutic efficacy of osimertinib in the subcutaneous and orthotopic NSCLC mouse models, enabling effective tumor suppression at a reduced dose and frequency upon *i.v.* administration.

## Results

### Preparation and characterization of RNP-SA-RBC

**Figure 2:**
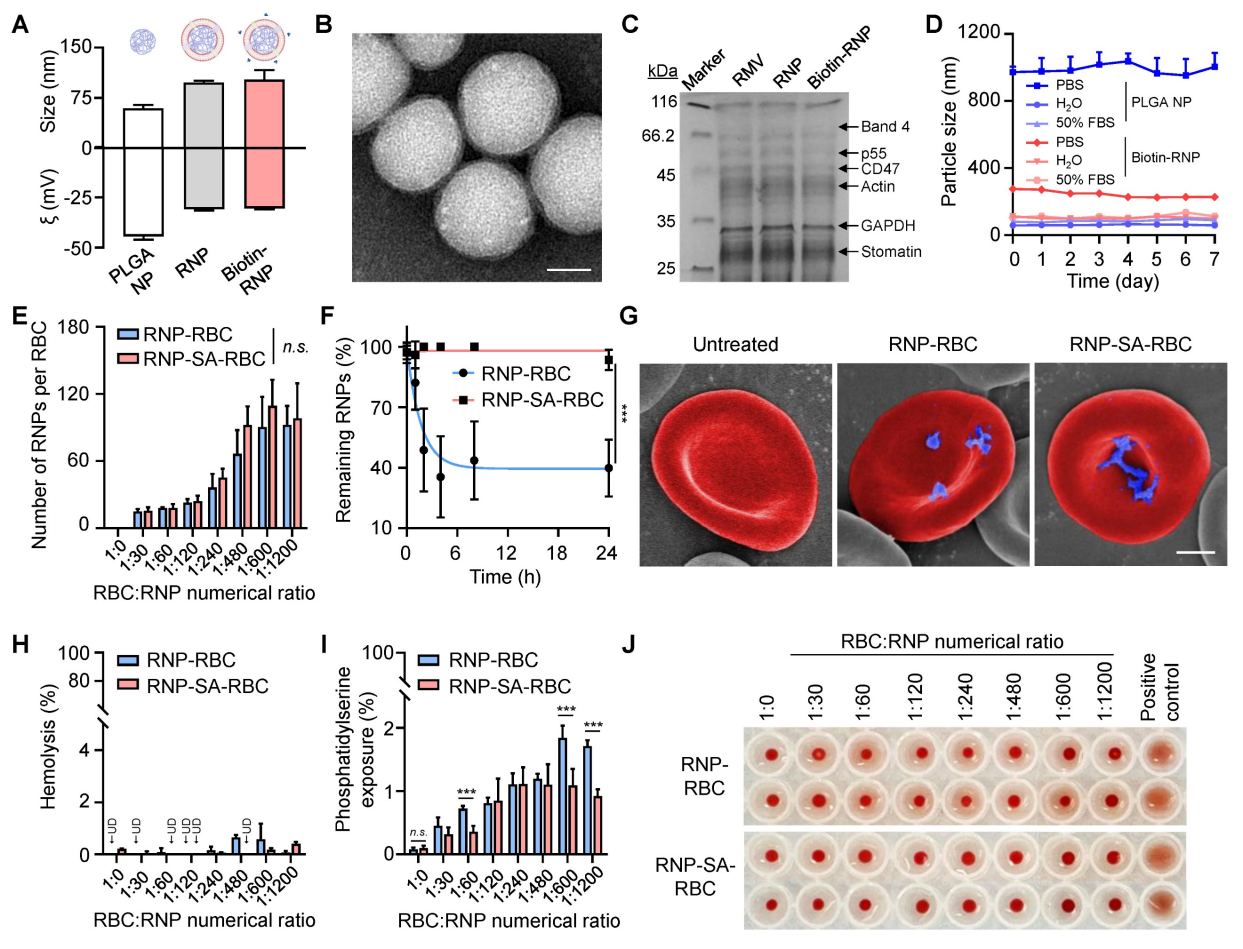
** Preparation and characterization of RNP-SA-RBC.** (**A**) Size and surface zeta potential (ξ) measurements of PLGA core (NP), RNP, and biotin-RNP. (**B**) TEM imaging of biotin-RNP. Scale bar: 40 nm. (**C**) SDS-PAGE protein analysis of RBC membrane vesicle (RMV), RNP and biotin-RNP. (**D**) Stability of NP and biotin-RNP in PBS, H_2_O, and 50% FBS over 7 days. (**E**) The estimated numbers of biotin-RNP loaded per RBC or SA-RBC at different RBC:RNP numerical ratios. (**F**) Time-dependent release of NPs in the RNP-RBC group and RNP-SA-RBC group over 24 h. (**G**) Pseudo-colored SEM images of untreated RBC, RNP-RBC and RNP-SA-RBC. Scale bar: 1 µm. (**H**-**J**) Quantification of hemolysis (**H**), phosphatidylserine (PS) exposure (**I**), and agglutination levels (**J**) in the RNP-RBC and RNP-SA-RBC at different RBC:RNP numerical ratios. RBCs with phytohemagglutinin (PHA, 100 µg/mL) treatment was used as a positive control. UD: undetected.

In the study, the murine RBCs were first isolated from whole blood, and their membranes were extracted *via* hypotonic treatment. Meanwhile, the polymeric cores were synthesized using a nanoprecipitation method, wherein PLGA polymer that dissolved in acetone was injected into ultrapure water. RBC membranes were then coated onto the surface of PLGA cores under ultrasonication to prepare the RNP, which further modified with a biotinylated lipid (5 wt%) on the cell membrane to fabricate the biotin-RNP through a lipid post-insertion strategy. As shown in **Figure 2A**, dynamic light scattering (DLS) analysis revealed that the average size of RNP (98 ± 2.6 nm) and biotin-RNP (104.2 ± 17.2 nm) was larger than that of the uncoated PLGA NPs (59.4 ± 5.3 nm), confirming the coating of cell membrane on the surface of polymeric cores. Moreover, the surface zeta (ξ)-potential of both RNP (- 30.4 ± 0.4 mV) and biotin-RNP (- 30.9 ± 0.7 mV) was less negative compared to the PLGA NPs (- 44.4 ± 1.6 mV), mainly due to the charge screening effect after the membrane coating. Additionally, transmission electron microscopy (TEM) images of biotin-RNP revealed a well-defined spherical core-shell structure, with no apparent structural alterations after surface biotinylation compared to the RNPs (**Figure 2B** and **S1**). These observations further validate the successful cloaking of the polymeric cores with the RBC membrane. The membrane protein profiles of RNP and biotin-RNP were also characterized using sodium dodecyl sulfate polyacrylamide gel electrophoresis (SDS-PAGE). Compared with emptied RBC membrane vesicle (RMV), RNP and biotin-RNP retained almost all of the key membrane proteins on the RBCs reported previously, including the band 4, p55, CD47, actin, and stomatin (**Figure 2C**). Unlike bare PLGA NPs which aggregated rapidly in phosphate-buffered saline (PBS), biotin-RNP exhibited consistent hydrodynamic sizes over 7 days in PBS, water, and 50% fetal bovine serum (FBS), revealing their excellent colloidal stability in these buffer solutions (**Figure 2D**). These data demonstrated the successful fabrication of biotin-RNP.

The RBC hitchhiking strategy, which involves the non-specific adsorption of NPs onto the RBC surface, is a common method for external drug loading. This is typically achieved through simple co-incubation, allowing for passive attachment. However, this nonspecific interaction may lack stability upon intravenous injection. To evaluate the feasibility and potential advantages of a more stable attachment method, we compared conventional hitchhiking with a high-affinity bioconjugation strategy. Accordingly, we developed the RBC-conjugated RNP (RNP-SA-RBC) *via* a simple, mild and specific biotin-SA approach. Specifically, isolated murine RBCs were functionalized with biotin N-hydroxysuccinimide ester (biotin-NHS) through amide condensation with membrane protein amine groups. These biotinylated RBC were then conjugated with SA to generate SA-RBCs. Subsequent incubation with biotin-RNP enabled surface conjugation *via* high-affinity biotin-SA interactions, yielding the stable RNP-SA-RBC complex. To optimize conjugation efficiency, the biotin-RNP was pre-labeled with a hydrophobic fluorophore (DiR) encapsulated within the polymeric cores, and then incubated with either SA-RBC or RBC at varying numerical ratios. After removing unbound NPs, the RNP-loaded RBCs were lysed, and the amount of bound RNP was quantified by measuring the near-infrared fluorescence of DiR at 780 nm. As shown in **Figure 2E**, the numbers of biotin-RNP bound to RBCs increased gradually with the addition of biotin-RNP and reached a plateau (~110 RNP per RBC) at an RBC:RNP numerical ratio of 1:600. Moreover, the number of biotin-RNP on RBCs surface showed no significant difference between the RNP-RBC (by adsorption) and RNP-SA-RBC (by conjugation), implying that the nonspecific adsorption may also contribute to the initial binding of biotin-RNP to RBCs. Interestingly, after 24 h storage in 1× PBS (pH 7.4, 5 mM glucose) at 37 °C, we observed a negligible dissociation of bound biotin-RNP in the RNP-SA-RBC group (8.9% release). On the contrary, the RNP-RBC group exhibited significant NP detachment (69.8% release) (**Figure 2F**). Given that the tumor microenvironment is a complex and dynamic niche that constantly subjected to physical and mechanical pressures, we further evaluated the stability of RNP-SA-RBC under elevated interstitial fluid flow and high vascular permeability, which generate interstitial shear stress (approximately 0.1 to 1 dyn/cm^2^) 50, 51. Accordingly, the shear stability of RNP-RBC and RNP-SA-RBC was assessed under physiologically relevant conditions corresponding to approximately 1 dyn/cm², simulating interstitial fluid flow at the tumor site. As demonstrated in **Figure S2**, shear-induced detachment of RNPs from RNP-SA-RBC was markedly lower than that from RNP-RBC. A significantly higher proportion of NPs remained bound to the RBC surface in the conjugated group (61.4%) compared to nonspecific adsorption group (40.3%), demonstrating the superior stability of biotin-SA-mediated conjugation over passive adsorption for RNP attachment to RBCs. Scanning electron microscopy (SEM) images further confirmed that a greater number of biotin-RNP were efficiently localized on the RBC surface in the RNP-SA-RBC group compared to the RNP-RBC group, without causing noticeable morphological alterations relative to healthy RBCs (**Figure 2G**). These results confirmed the effective, stable, and safe conjugation of biotin-RNP onto RBCs surface.

We subsequently evaluate the hematocompatibility of biotin-RNP following their binding to RBCs. Hemolysis levels serve as a major indicator to assess the safety of NPs on RBCs, as membrane disruption may cause physiological side effects during transfusion, potentially triggering undesired immune responses or vascular complications 38, 52. As shown in **Figure 2H**, negligible hemolysis (< 1%) was observed on RNP-loaded RBCs at all of the tested RBC:RNP numerical ratios during the binding process. However, after 24 h of incubation, the RNP-RBC induced a higher hemolysis rate (2.5%) than that of RNP-SA-RBC (0.3%), implying an enhanced blood safety through biotin-SA conjugation process (**Figure S3**). Moreover, the translocation of phosphatidylserine (PS) to the outer membrane leaflet of RBCs serves as another marker for cell senescence and damage, which can trigger the clearance of RBCs from blood circulation and negatively impact drug delivery efficacy 37. Thus, the extent of PS exposure in RNP-loaded RBCs was also analyzed by flow cytometry using Annexin V-FITC, a dye-labeled protein that specifically recognize the PS on the cell membrane. As shown in **Figure 2I**, a moderate elevation of PS exposure (< 2%) on RBCs was determined with the increasing of RBC:RNP numerical ratios in both RBCs and SA-RBCs, suggesting a dose-dependent RBC damage by biotin-RNP. Importantly, RBCs in the RNP-SA-RBC group exhibited significantly lower PS exposure compared to the RNP-RBC group, indicating that surface modification of RBCs* via* biotin-SA conjugation can minimize the possibility of eryptosis compared to passive adsorption. Additionally, RBCs agglutination, a pathological phenomenon where RBCs clump into aggregates and potentially cause vascular occlusion, tissue ischemia, and endothelial dysfunction, was assessed using a gold-standard round-bottom-well assay 53. In this assay, non-aggregated RBCs form a compact dot at the bottom of the well, while aggregated RBCs spread into a film. Phytohemagglutinin (PHA) was used as a positive control to induce 100% agglutination of RBCs. As shown in **Figure 2J**, minimal agglutination was observed in RNP-RBC group at RBC:RNP numerical ratios up to 1:1200, while no significant RBC aggregation was detected in RNP-SA-RBC groups at any of the tested ratios. Based on these findings, an optimal RBC:RNP numerical ratio of 1:600 was used for subsequent studies. To assess the intrinsic biocompatibility of the RNP-SA-RBC, its cytotoxicity was further evaluated in healthy cells. As shown in **Figure S4**, exposure to RNP-SA-RBC resulted in negligible cytotoxicity toward both endothelial cells (HUVEC) and fibroblasts (NIH/3T3 cells), indicating minimal carrier-induced cytotoxicity. These results confirm that the biotin-SA conjugation and RBC surface modification did not adversely affect cellular viability, demonstrating the good *in vitro* biocompatibility of RNP-SA-RBC. Taken together, these studies suggested that the biotin-SA conjugation approach offers a safer and more reliable strategy for RNP loading onto RBCs.

### Enhanced blood circulation and tumor accumulation of RBC-conjugated RNP *in vivo*

**Figure 3:**
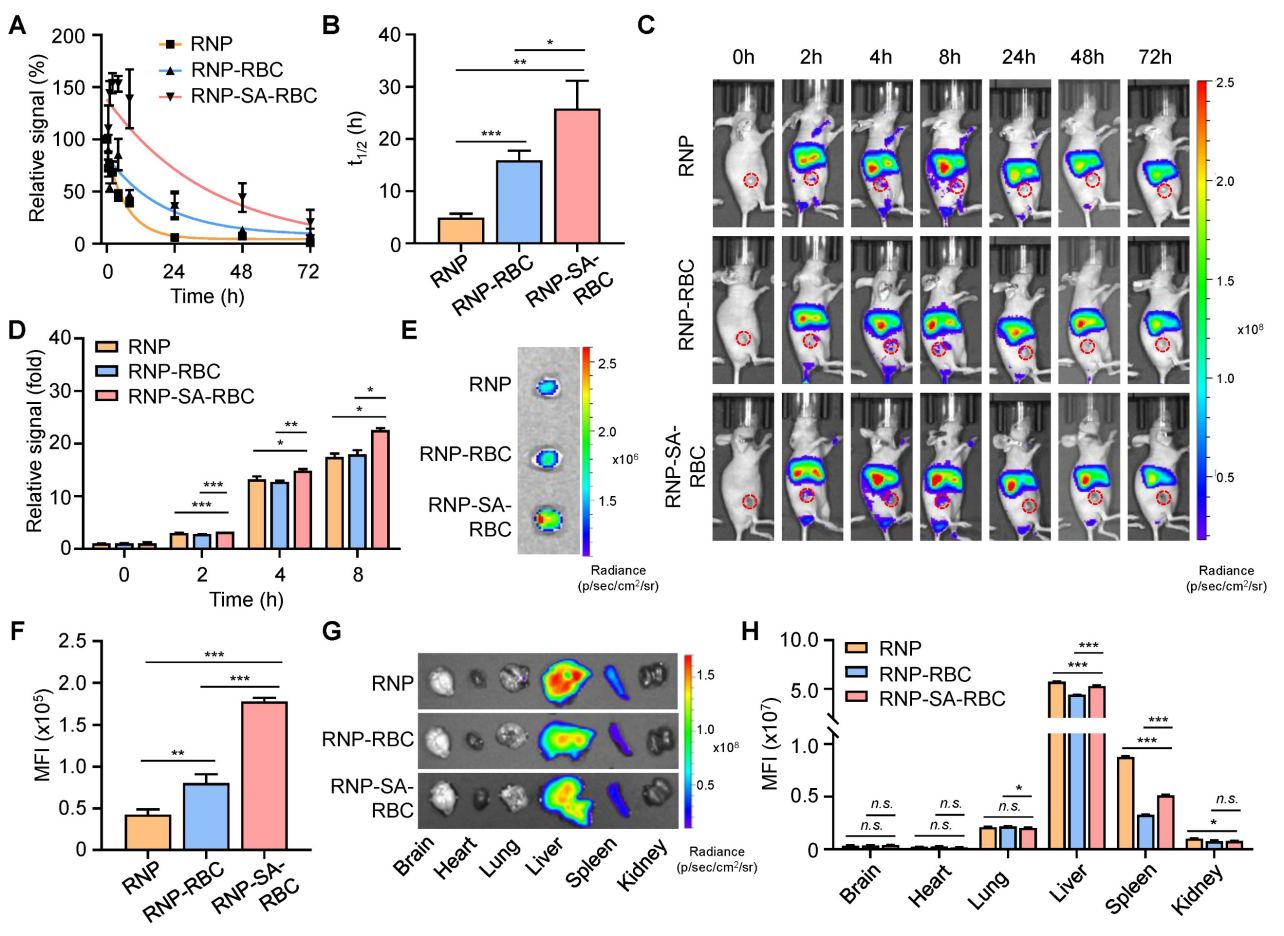
** Enhanced blood circulation and tumor accumulation of RBC-conjugated RNPs *in vivo*.** (**A-B**) Relative fluorescence signals (**A**) and serum half-lives (**B**) of DiR-labeled RNP, RNP-RBC and RNP-SA-RBC (20 mg/kg) at different time points post-injection. (**C-D**) *In vivo* imaging of PC9/ER tumor-bearing mice (**C**) and relative signals of tumors (**D**) at different time points after *i.v.* injection of DiR-labeled RNP, RNP-RBC and RNP-SA-RBC. Red circles indicate the tumor regions in mice. (**E-F**) Representative PC9/ER tumor images (**E**) and mean fluorescence intensity (MFI) of tumors (**F**) harvested from mice at 8 h post-injection of DiR-labeled RNP, RNP-RBC and RNP-SA-RBC. (**G-H**) *Ex vivo* imaging (**G**) and relative fluorescence signals (**H**) of major organs (e.g., brain, heart, lung, liver, spleen, and kidneys) at 8 h post-injection of different groups in tumor-bearing mice.

To evaluate the blood circulation properties of RBC-loaded RNP *in vivo*, Balb/c mice were *i.v.* injected with DiR-labeled RNP, RNP-RBC, and RNP-SA-RBC (20 mg/kg, *n* = 3), respectively. Blood samples were then collected at different time points for fluorescence measurements. As shown in **Figure 3A**, these formulations displayed a nonlinear elimination curve in a time-dependent manner. The apparent half-lives (denoted as the time required for 50% of the targets being cleared) of RNP, RNP-RBC, and RNP-SA-RBC in the blood were determined to be 4.9 ± 0.7 h, 15.9 ± 1.8 h, and 25.8 ± 5.3 h, respectively (**Figure 3B**). Compared to the RNP-RBC, the relative short circulation profile of RNP is likely attributed to its inability to evade rapid clearance by the RES *in vivo*, whereas loading onto the RBC surface improved circulation. A further enhanced blood retention time was observed in the RNP-SA-RBC group, which can be ascribed to the stable biotin-SA conjugation between RNPs and RBCs, and consistent strategy for NP loading onto RBCs. We also studied the tumor accumulation capability of RBC-conjugated RNPs in tumor-bearing mice. In this study, a human NSCLC (PC9/ER) cell line were subcutaneously implanted into the Balb/c nude mice to establish xenograft lung tumor model. When the tumors volume reached approximately 50 mm^3^, the mice were *i.v.* administrated with DiR-labeled RNP, RNP-RBC, and RNP-SA-RBC (20 mg/kg, *n* = 3), followed by the fluorescence imaging of these animals at designated time points. As illustrated in **Figure 3C**, the fluorescence signal of all groups at the tumor site elevated gradually over time, reaching saturation at 8 h post-injection. When compared to the RNP and RNP-RBC-treated groups, RNP-SA-RBC showed the highest fluorescence signal in tumors over an 8-h period (**Figure 3D**). To confirm these observations, the tumors were harvested from mice at 8 h post-injection for *ex vivo* imaging. The mean fluorescence intensity (MFI) of the tumors treated with RNP-RBC and RNP-SA-RBC was 1.2-fold and 3.2-fold higher, respectively, than that of the RNP group (**Figure 3E** and **F**). The enhanced tumor accumulation of RBC-conjugated RNP might be attributed to the prolonged circulation time through stable biotin-SA conjugation and the abnormal permeability of tumor vasculature that facilitate the preferential retention of RNP within the tumor microenvironment. Given the enhanced tumor accumulation observed *in vivo*, the cellular uptake mechanism of RNPs in tumor cells was next elucidated by employing specific endocytosis inhibitors to assess the contribution of each pathway. Uptake of RNPs was markedly reduced in both cell lines pretreated with chlorpromazine (CPZ) or methyl-β-cyclodextrin (MβCD), whereas no significant change was observed in cells treated with amiloride hydrochloride (AH) (**Figure S5**). These results indicate that RNP internalization in tumor cells occurs predominantly through clathrin-mediated and caveolae/raft-dependent endocytic pathways. Moreover, major organs (*e.g.*, brain, heart, lung, liver, spleen, and kidneys) were also harvested at 8 h post-injection for imaging studies. As shown in **Figure 3G** and **H**, RNP, RNP-RBC and RNP-SA-RBC were predominantly distributed in the liver rather than the spleen, which are the two primary organs of the RES for NP clearance from the body. Together, RBC- conjugated RNPs demonstrate an improved circulation profile and tumor accumulation, indicating their potential advantages for enhanced drug delivery and tumor therapy.

### Antitumor efficacy of drug-loaded RNP in lung tumor cells

**Figure 4:**
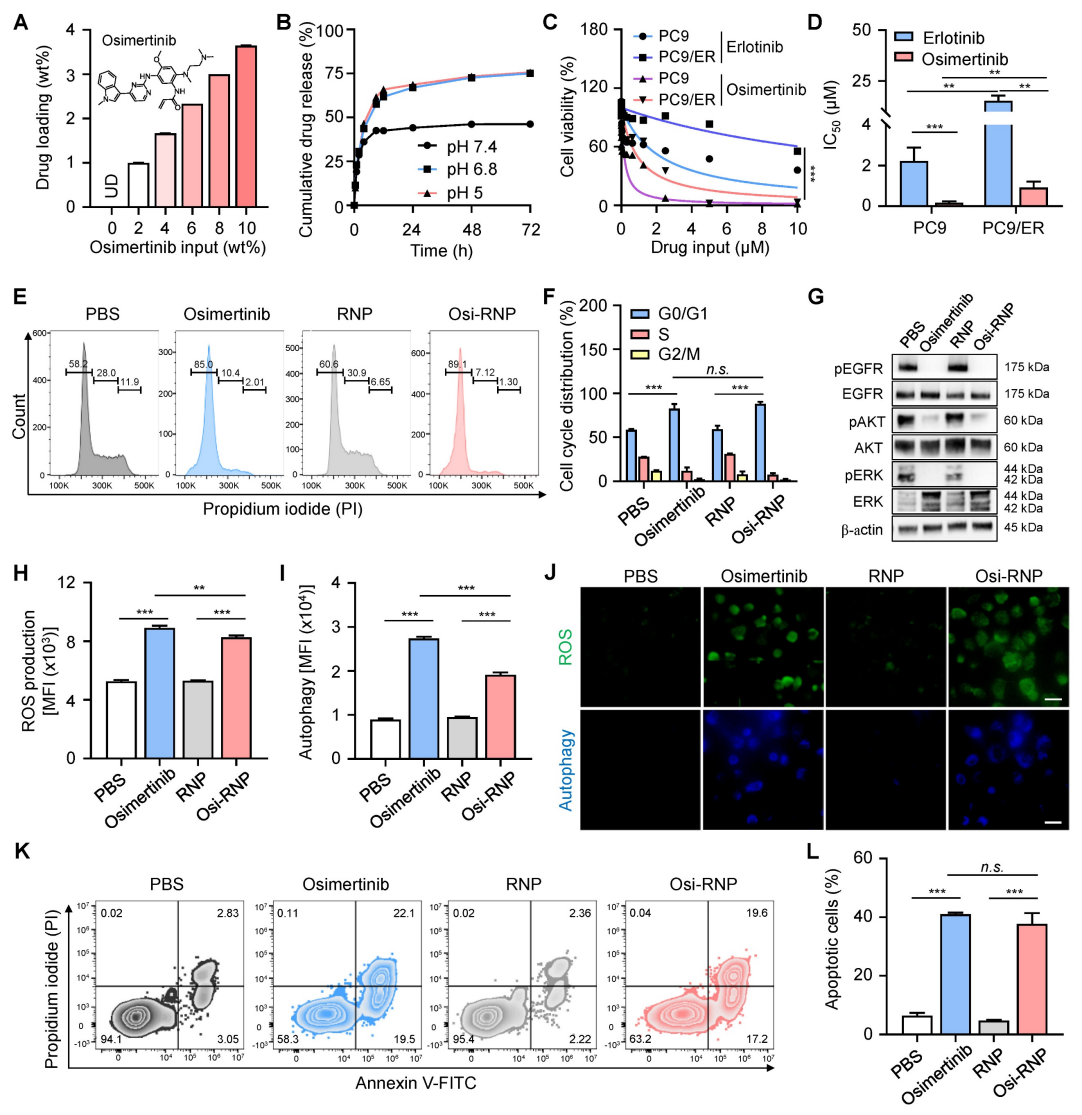
** Mechanisms of osimertinib-loaded RNPs in inhibiting the proliferation of lung tumor cells.** (**A**) Quantification of drug loading in RNPs with increasing osimertinib input to RNP weight ratios. (**B**) Cumulative drug release profile of osimertinib from RNP in PBS at pH 7.4, pH 6.8, and pH 5.0 at 37 °C over 72 h. (**C**-**D**) Cell viability (**C**) and half-maximal inhibitory (IC_50_) concentrations (**D**) of PC9 and PC9/ER cells incubated with erlotinib and osimertinib at different concentrations for 72 h. (**E-F**) Flow cytometry analysis of the cell cycle (**E**) and the percentage of cell populations (**F**) arrested at G0/G1, S and G2/M phases upon exposure of osimertinib, RNP and Osi-RNP at the same drug dosage (0.5 µM). (**G**) Western blot analysis of EGFR signaling in different groups. (**H**-**I**) MFI of ROS production (**H**) and autophagy formation (**I**) in the PC9/ER cells treated with osimertinib, RNP and Osi-RNP. (**J**) Fluorescence microscopy images of ROS production and autophagy formation in the PC9/ER cells treated with osimertinib, RNP and Osi-RNP. (**K-L**) Flow cytometric assessment of apoptosis (**K**) and the percentage of apoptotic cells (**L**) by the Annexin V-FITC/PI staining assay in drug-treated PC9/ER cells.

Next, we investigated the antitumor efficacy of the drug and its underlying therapeutic mechanisms in the PC9/ER cell line. Osimertinib, a third-generation EGFR-TKI widely used in clinical settings, is recognized for its potent and selective inhibition of EGFR-sensitizing mutations (*e.g.*, T790M) and is commonly employed to treat patients who develop resistance following first- or second-generation EGFR-TKI therapies. In this study, the hydrophobic osimertinib was first encapsulated into both the polymeric cores and the phospholipid bilayer of RNP (denoted “Osi-RNP”), and the drug loading capacity and release profiles were then quantified using HPLC based on a standard curve of osimertinib at known concentrations (0-1 µg/mL) (**Figure S6**). As shown in **Figure 4A**, the loading amounts of osimertinib in RNP correlated positively with increasing levels of initial drug inputs. Nevertheless, when the input ratio exceeded 10 wt%, notable aggregation of RNP was observed (**Figure S7**). Therefore, we selected a drug input of 10 wt% with a maximal drug loading rate of 3.6 ± 0.01 wt% for the subsequent studies. We further evaluated the drug release profile of Osi-RNP in 1× PBS (pH 7.4). As shown in **Figure 4B**, osimertinib release from Osi-RNPs exhibited a pH-dependent profile. At physiological pH (pH 7.4), the release profile displayed an early burst of approximately 42% within the first 8 h, followed by a prolonged release (~48%) over a period of 72 h. Under mildly acidic (pH 6.8) and lysosomal (pH 5.0) conditions, the cumulative release increased to 57.5% and 61.6% at initial 8 h, respectively, and further reached about 75% by 72 h. These results indicate enhanced drug release at lower pH, consistent with the acidic tumor microenvironment and intracellular compartments. The release kinetics was also analyzed using the diffusion-dominant Higuchi model: *M_t_ = K*t*^1/2^*, where *M_t_* represents the amount of drug released at time t, and *K* is the Higuchi constant, which characterize the drug release rate as a function of time (**Figure S8**) 25. Plotting the cumulative percentage of osimertinib released against the square root of time resulted in a strong linear correlation (R^2^ = 0.96, 0.98 and 0.98) under different pH conditions. The curve fitting exhibited a diffusion-controlled release mechanism in this nanoplatform. Based on the Higuchi model, the release constant was calculated as 15.7 ± 0.4 h^-1/2^, 19.8 ± 0.1 h^-1/2^, 21.7 ± 0.1 h^-1/2^ at pH 7.4, pH 6.8 and pH 5.0, respectively.

We then assessed the drug resistance profile of PC9/ER cells in comparison to their parental, non-drug-resistant NSCLC counterpart, PC9 cells. Both cell lines were exposed to equivalent concentrations of free erlotinib or osimertinib for 72 h, followed by the assessment of cell viability. As shown in **Figure 4C**, the cell viability of both PC9/ER and PC9 cells decreased in a dose-dependent manner upon treatment with erlotinib and osimertinib. At all tested concentrations of erlotinib, PC9/ER cells remained higher viability than the PC9 cells due to the presence of the T790M mutation in the PC9/ER cells, which is a well-known gatekeeper mutation that hinders the specific binding between erlotinib and EGFR 54. The half-maximal inhibitory concentration (IC_50_) value of osimertinib-treated PC9/ER cells (0.9 ± 0.3 µM) and PC9 cells (0.2 ± 0.1 µM) was greatly lower compared to erlotinib-treated PC9/ER cells (19.7 ± 5.4 µM) and PC9 cell (1.8 ± 0.5 µM), respectively (**Figure 4D**). Notably, the apoptosis assay demonstrated that osimertinib exerted enhanced cytotoxicity in both PC9/ER and PC9 tumor cells (**Figure S9**). This effect is attributed to its selective targeting of EGFR mutants (*e.g.*, T790M) while sparing the inhibition activity of wild-type EGFR, thereby reducing toxicity and enabling long term treatment of NSCLC 54. Additionally, NSCLC tumorigenesis is often driven by EGFR mutations, amplification, or overexpression, which lead to activation of multiple downstream EGFR signaling cascades, most notably the mitogen-activated protein kinase (MAPK)/extracellular signal-regulated kinases (ERK) and phosphatidylinositol 3-kinase (PI3K)/AKT pathways 55, 56. EGFR activation triggers RAS-MEK-ERK signaling to promote tumor cell proliferation. In parallel, activation of the PI3K pathway results in AKT phosphorylation, which plays a central role in regulating cell survival and apoptotic processes. Accordingly, western blot analysis was further employed to examine the phosphorylation levels of EGFR in tumor cells, along with the activation status of several key downstream signaling molecules, including AKT in the PI3K/AKT pathway, and ERK in the MAPK/ERK pathway. As shown in **Figure S10**, upon erlotinib treatment, the levels of phosphorylated kinases (*e.g.*, pEGFR, pAKT, pERK) exhibited a significant decrease in PC9 cells but remained unchanged in PC9/ER cells when compared to PBS-treated groups, indicating the drug-resistant properties of PC9/ER cells. In contrast, osimertinib treatment led to a pronounced downregulation of these phosphorylated proteins in both PC9 and PC9/ER cells, demonstrating its efficacy in overcoming resistance and enhancing therapeutic outcomes in lung tumor cells.

We next extended the evaluation of the antitumor efficacy of free osimertinib and Osi-RNP at the same drug dosages in PC9/ER cells. The cytotoxicity of PC9/ER cells upon Osi-RNP treatment was comparable to the cells treated with osimertinib at all tested concentrations (0-5 µM) (**Figure S11**), while RNP alone exhibited negligible impact on the cell viability of PC9/ER cells (**Figure S12**). To investigate the potential mechanisms of PC9/ER cell death, we first performed the cell cycle studies in tumor cells using the propidium iodide (PI) staining. As shown in **Figure 4E** and** F**, the proportion of cells arrested in the G0/G1 phase was significantly higher in the osimertinib (82.6%) and Osi-RNP (88.1%) treated groups than that in the PBS (58.4%) and RNP (59.3%) treated cells. Meanwhile, the percentages of cells arrested at S and G2/M phases were reduced in the drug-treated groups compared to the controls. These results suggest that Osi-RNP inhibits the proliferation of PC9/ER cells by promoting G0/G1 phase arrest and impeding cell cycle progression. Furthermore, western blot results further revealed a marked downregulation of pEGFR, pAKT and pERK in PC9/ER cells following treatment with either free osimertinib or Osi-RNP (**Figure 4G**). This inhibition of cell proliferation can be attributed to the suppression of PI3K/AKT and MAPK/ERK signaling pathways, which are known to regulate the activation of cell cycle-related transcription factors and proteins that essential for G1-to-S phase progression, as well as anti-apoptotic cascade signals 56. Moreover, the intracellular reactive oxygen species (ROS) levels and ROS-induced autophagy were assessed by flow cytometry using dihydroethidium (a fluorogenic probe of superoxide radicals) and monodansylcadaverine (a fluorescent probe for autophagosome labeling). As shown in **Figure 4H** and **I**, ROS and autophagy levels were significantly elevated in PC9/ER cells treated with osimertinib or Osi-RNP compared to control groups. Similar observations were also captured by fluorescence microscopy images (**Figure 4J**). Additionally, drug-induced apoptosis was evaluated using an Annexin V-FITC and PI staining assay. As shown in **Figure 4K** and** L**, the percentage of early apoptotic (Annexin V^+^/PI^-^) and late apoptotic (Annexin V^+^/PI^+^) cells was comparable between the osimertinib and Osi-RNP-treated groups, which were considerably higher than the PBS and RNP-treated control groups. Overall, these findings demonstrated that osimertinib-loaded RNP exhibit potent antitumor activity by inhibiting cell cycle progression and multiple signaling pathways, while concurrently augmenting ROS production and autophagy level, resulting in the induction of apoptosis in lung tumor cells.

### *In vivo* antitumor efficacy of RBC-conjugated Osi-RNP in subcutaneous xenograft model

**Figure 5:**
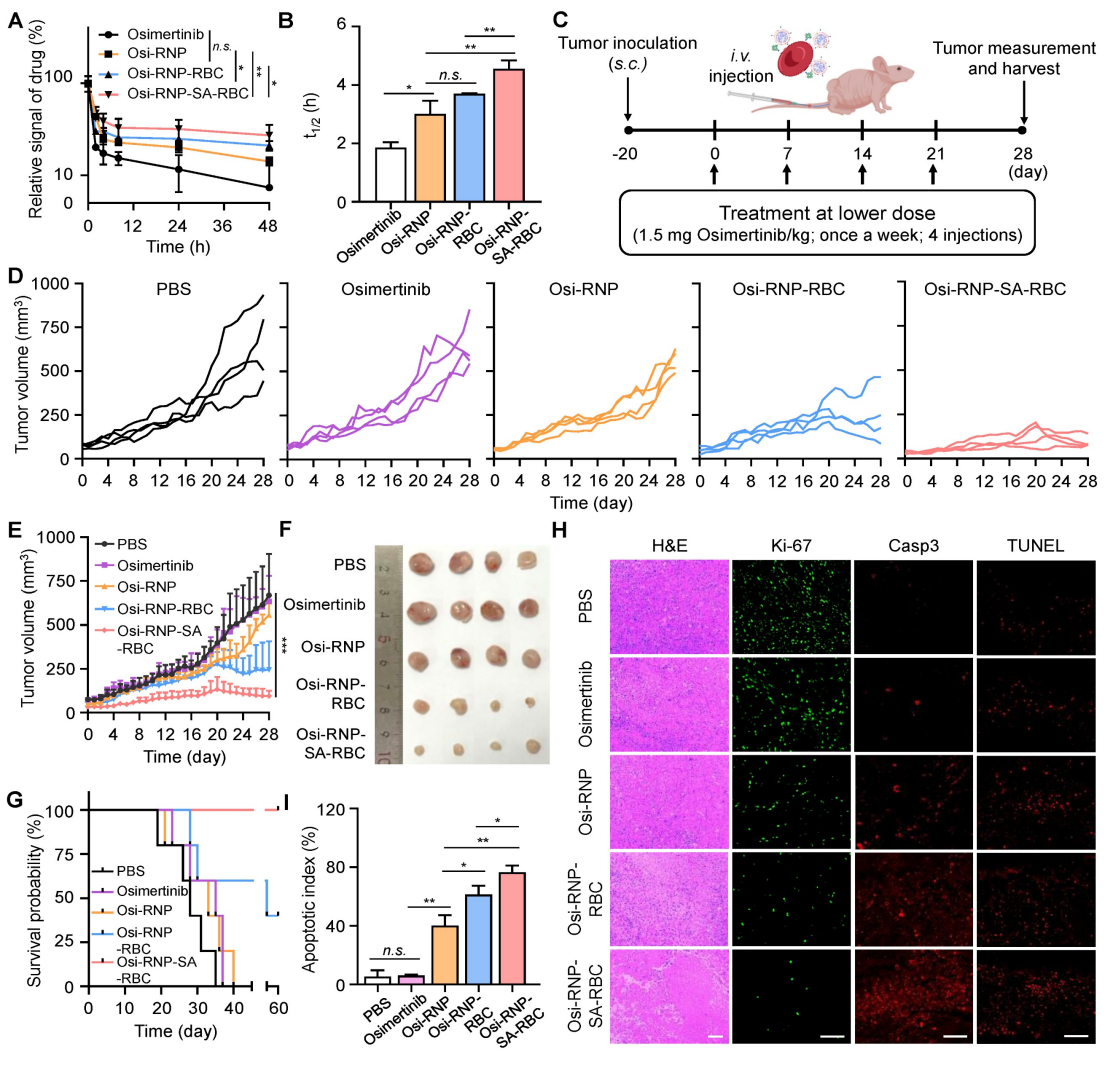
** Enhanced pharmacokinetics and antitumor efficacy of osimertinib-loaded RNP-SA-RBC in a subcutaneous drug-resistant tumor model *in vivo*.** (**A-B**) Pharmacokinetics (**A**) and elimination half-lives (**B**) of osimertinib, Osi-RNP, Osi-RNP-RBC and Osi-RNP-SA-RBC upon *i.v.* injection in PC9/ER tumor-bearing mice. The statistical annotations indicate comparisons performed at the 48-h time point. (**C**) Schematic illustration of the treatment timeline for *in vivo* study. (**D-E**) Individual (**D**) and average (**E**) tumor growth curves of tumor-bearing mice after treated with PBS, osimertinib, Osi-RNP, Osi-RNP-RBC, and Osi-RNP-SA-RBC (1.5 mg/kg, once a week for four doses; *n* = 4). (**F**) PC9/ER xenograft tumor images collected from mice at the endpoint of different therapeutic regimens. (**G**) Kaplan-Meier survival analysis of tumor-bearing mice after intravenous administration of different treatments, with survival monitored for 60 days (*n* = 5). (**H**) Representative tumor section images showing H&E staining, Ki-67 cell proliferation analysis, caspase-3 (casp3) immunostaining, and TUNEL assay from various groups. Scale bar: 100 µm. (**I**) Quantitative analysis of apoptosis index determined by combined evaluation of Caspase-3 staining in tumor tissues from different treatment groups at day 28.

Before evaluating the therapeutic efficacy of osimertinib-loaded RNP-SA-RBC (termed as Osi-RNP-SA-RBC) *in vivo*, we first studied the pharmacokinetic profiles of free osimertinib and osimertinib-loaded platform (RNP, RNP-RBC and RNP-SA-RBC) upon tail vein injection in mice, followed by blood samples collection at predetermined time points for HPLC analysis. As shown in **Figure 5A**, all groups showed a nonlinear and time-dependent elimination curve over a period of 48 h. Based on the classical two-compartment model in pharmacokinetic model, the elimination half-lives of osimertinib, Osi-RNP, Osi-RNP-RBC and Osi-RNP-SA-RBC groups in the blood were determined as 1.9 ± 0.2 h, 3.0 ± 0.5 h, 3.7 ± 0.02 h, and 4.6 ± 0.3 h, respectively (**Figure 5B**). When compared to the osimertinib-treated mice, the area under the curve (AUC) in the Osi-RNP-SA-RBC-treated group (2.4-fold increase) was much higher than that in the Osi-RNP (1.6-fold increase) and Osi-RNP-RBC (1.9-fold increase) (**Figure S13**). These results reveal an improved pharmacokinetics profile of the EGFR-TKI after encapsulation into the biomimetic RNP and further conjugation with RBCs.

Subsequently, we determined the therapeutic efficiency of osimertinib-loaded RNP-SA-RBC using a xenograft tumor model of NSCLC. In preclinical studies, osimertinib is commonly treated in tumor-bearing mice through oral administration daily at doses ranging from 5 to 25 mg/kg (25 mg/kg approximates the clinically approved 80 mg dose daily in NSCLC patients) 57, 58. Given the enhanced pharmacokinetics properties of osimertinib after loading in RNP-SA-RBC, we hypothesized that a reduced dosage and less frequent administration may enable efficacious treatment outcomes of NSCLC tumor model. To test this hypothesis, the PC9/ER tumor-bearing Balb/c nude mice received a total of four* i.v.* injections of free osimertinib, Osi-RNP, Osi-RNP-RBC and Osi-RNP-SA-RBC at a lower dosage (1.5 mg/kg), administrated once per week (**Figure 5C**). As shown in **Figure 5D** and** E**, substantial tumor suppression was observed in the Osi-RNP-SA-RBC group in a time-dependent manner. When compared to the PBS-treated mice at day 28, the inhibition rate of tumor growth was determined upon osimertinib (5.1% decrease), Osi-RNP (16.7% decrease), Osi-RNP-RBC (55.6% decrease), and Osi-RNP-SA-RBC (85.9% decrease) treatment at the same drug dosages, respectively (**Figure S14**). The antitumor efficacy was also further validated by examining the images of excised tumors, as well as the average tumor weight at the experimental end-point on day 28 (**Figure 5F** and **Figure S15**), which were significantly lower in the mice treated with Osi-RNP-SA-RBC than that in other control groups, while no significant body weight loss was observed in the tumor-bearing mice throughout the treatment period (**Figure S16**). In additional experiments, tumor-bearing mice administered free osimertinib at a clinically relevant high dose (25 mg/kg) *via* oral gavage exhibited less tumor growth suppression than those treated with Osi-RNP-SA-RBC (**Figure S17**). To evaluate the durability of the therapeutic effect, animal survival was monitored for 60 days post-initial treatment. Kaplan-Meier analysis revealed significant differences in survival outcomes between treatment groups (**Figure 5G**). Mice treated with Osi-RNP-SA-RBC exhibited the highest survival rate, with 100% surviving at the 60-day endpoint. Collectively, these results highlight the efficacy of the RBC-mediated delivery platform in improving therapeutic outcomes.

The tumor tissues from each group were further examined using hematoxylin and eosin (H&E) staining, Ki-67 immunofluorescence staining to evaluate cell proliferation, and caspase-3 and TUNEL immunofluorescence staining to determine apoptotic activity. As shown in **Figure 5H**, extensive necrotic regions were observed in the tumor tissue sections from mice treated with Osi-RNP-SA-RBC compared to other groups. Notably, the fluorescence signal of Ki-67 in the tumor tissues was significantly suppressed (96.6% decrease) upon Osi-RNP-SA-RBC treatment relative to PBS-treated mice. In contrast, only modest decreases in Ki-67 expression were determined in the Osi-RNP-RBC (85.2% decrease), Osi-RNP (72.1% decrease), and free osimertinib (3.2% decrease) groups (**Figure S18**). Additionally, apoptosis was evaluated to provide a comprehensive assessment of treatment efficacy. The activation of caspase-3, a key effector in apoptotic pathways, was measured and complemented by TUNEL staining to detect apoptotic DNA fragmentation. Immunohistochemical analysis revealed that Osi-RNP-SA-RBC treatment substantially increased apoptosis in tumor tissues relative to all other groups (**Figure 5H and S19**). The apoptotic index was significantly higher (95.7%) compared to the PBS (6.9%), Osi (7.7%), Osi-RNPs (50.3%) and Osi-RNP-RBCs (76.7%) (**Figure 5I**). Taken together, conjugating drug-loaded RNPs to RBCs enhances the pharmacokinetics profile of EGFR-TKI drug, facilitating improved drug delivery and substantially greater therapeutic efficacy against subcutaneous NSCLC mouse model at a reduced dose.

### Therapeutic performance in an orthotopic lung tumor model

**Figure 6:**
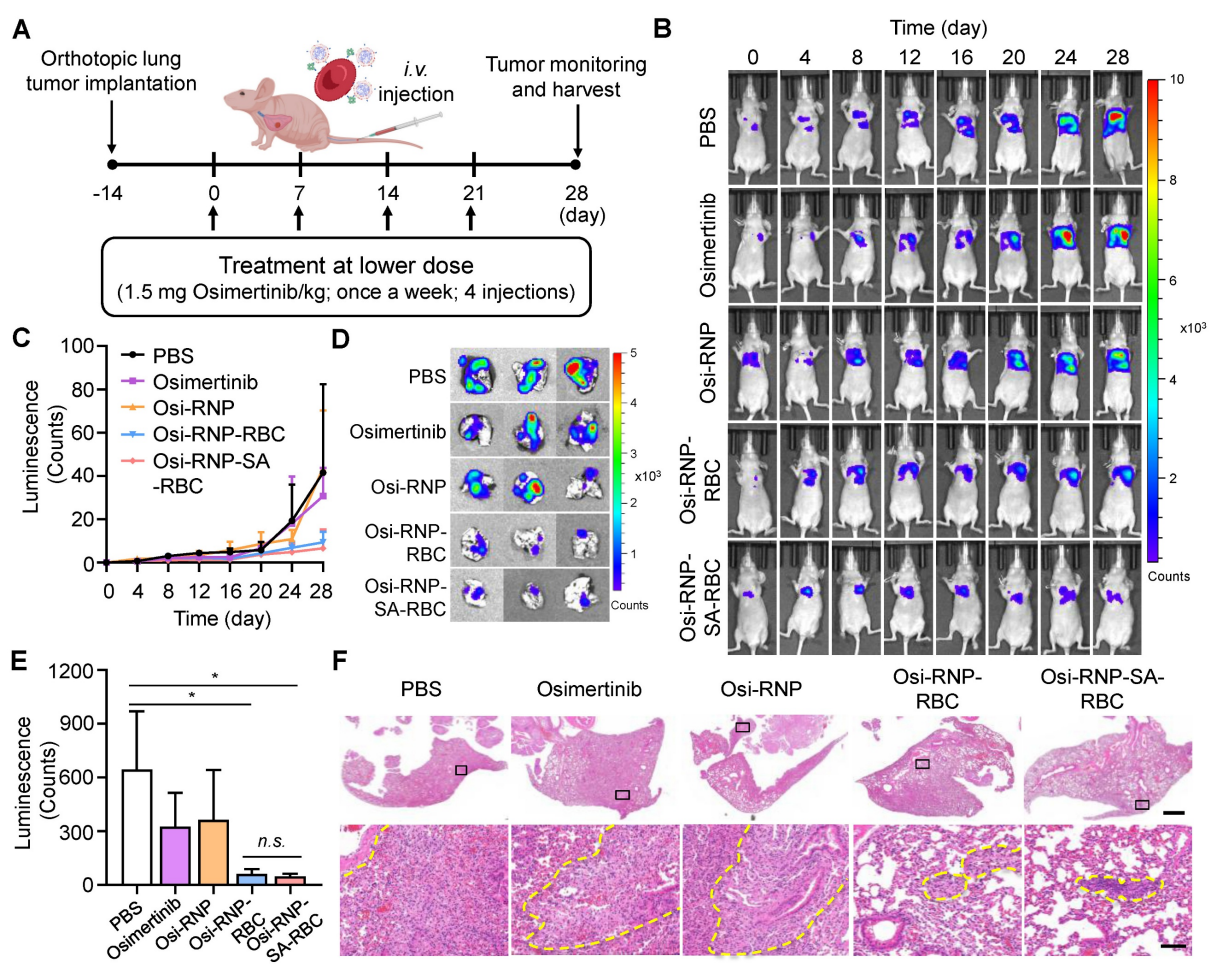
** Enhanced antitumor efficacy of osimertinib-loaded RNP-SA-RBC in an orthotopic lung tumor model *in vivo*.** (**A**) Schematic overview of the *in vivo* treatment schedule and experimental timeline. (**B-C**) *In vivo* bioluminescence imaging of A549-Luc orthotopic lung tumor-bearing mice (**B**) and corresponding quantitative analysis of lung tumor bioluminescence signals (**C**) at designated time points following *i.v.* injection of various treatment regimens (*n* = 3). (**D**-**E**) Representative images of excised lungs collected (**D**) and quantification of luminescence intensity of lungs (**E**) harvested at study endpoint. (**F**) Representative H&E-stained lung sections showing overall tissue morphology (Scale bar: 1 mm) and higher-magnification views (Scale bar: 100 µm). Tumor infiltration regions are outlined by yellow dotted lines.

Encouraged by the enhanced therapeutic efficacy observed in subcutaneous models, we next evaluated the antitumor potential of Osi-RNP-SA-RBC in an orthotopic lung tumor model. In this study, an orthotopic model was established by surgically exposing the left lung of Balb/c nude mice and injecting firefly luciferase-expressing A549 (A549-Luc) human NSCLC cells, followed by wound closure. The *in vitro* cytotoxicity of the formulation against A549-Luc cells was first confirmed and found to be comparable to free osimertinib (**Figure S20**), validating its relevance for this model. Following a two-week tumor establishment period, mice received weekly *i.v.* treatments at a low osimertinib dose (1.5 mg/kg) for four weeks (**Figure 6A**). Tumor progression was monitored *via* bioluminescence imaging every four days after intraperitoneal injection of D-luciferin. When compared to the PBS-treated mice at day 28, we found that the bioluminescence signals of A549-Luc tumor model were significantly decreased upon the treatment of Osi-RNP-RBC and Osi-RNP-SA-RBC at the same drug dosages (**Figure 6B** and **C**). The therapeutic outcomes in orthotopic model were further supported by a significant reduction in quantitative *ex vivo* luminescence intensity in excised lungs from different treatment groups. As shown in** Figure 6D** and **E**, compared to the PBS-treated mice, the inhibition rate of tumor growth was determined upon osimertinib (49.4% decrease), Osi-RNP (43.4% decrease), Osi-RNP-RBC (90.4% decrease), and Osi-RNP-SA-RBC (92.5% decrease), respectively. Although the Osi-RNP-SA-RBC group exhibited the lowest mean signal intensity, the difference relative to the Osi-RNP-RBC group was not statistically significant (*p* = 0.492). The comparable *in vivo* performance of Osi-RNP-RBC may be attributable to pulmonary retention of RNPs following partial desorption from RBCs, which could compensate for lower circulatory stability, thereby leading to a similar therapeutic efficacy comparable to that of Osi-RNP-SA-RBC. In addition, no significant body weight changes were observed across different groups (**Figure S21**). Collectively, these results indicate that stable RBC conjugation *via* biotin-SA interactions promote sustained drug delivery to lung tumors, enhancing *in vivo* antitumor efficacy.

Additionally, excised lungs were collected for histopathological analysis (**Figure S22**). As shown in **Figure 6F**, RBC-based treatment groups exhibited significant tumor suppression, with the most pronounced effect in the Osi-RNP-SA-RBC group. Histological examination of lung sections from Osi-RNP-SA-RBC-treated mice revealed substantial preservation of normal pulmonary architecture, characterized by largely intact alveolar structures and minimal residual tumor cell infiltration. In contrast, lungs from PBS-treated mice showed extensive tumor infiltration within the parenchyma, resulting in marked architectural disruption, where alveolar structures were obliterated and replaced by densely packed, pleomorphic tumor cells with high cellularity, and accompanied by prominent inflammatory infiltration. Although treatment with free osimertinib and Osi-RNPs partially reduced tumor burden relative to the PBS group, significant pulmonary structural disruption remained, reflecting incomplete tumor control. Together, these histopathological findings corroborate the superior antitumor efficacy and favorable safety profile of Osi-RNP-SA-RBC platform in the orthotopic lung tumor model.

### Biosafety assessment of drug-loaded RNP-SA-RBC *in vivo*

**Figure 7:**
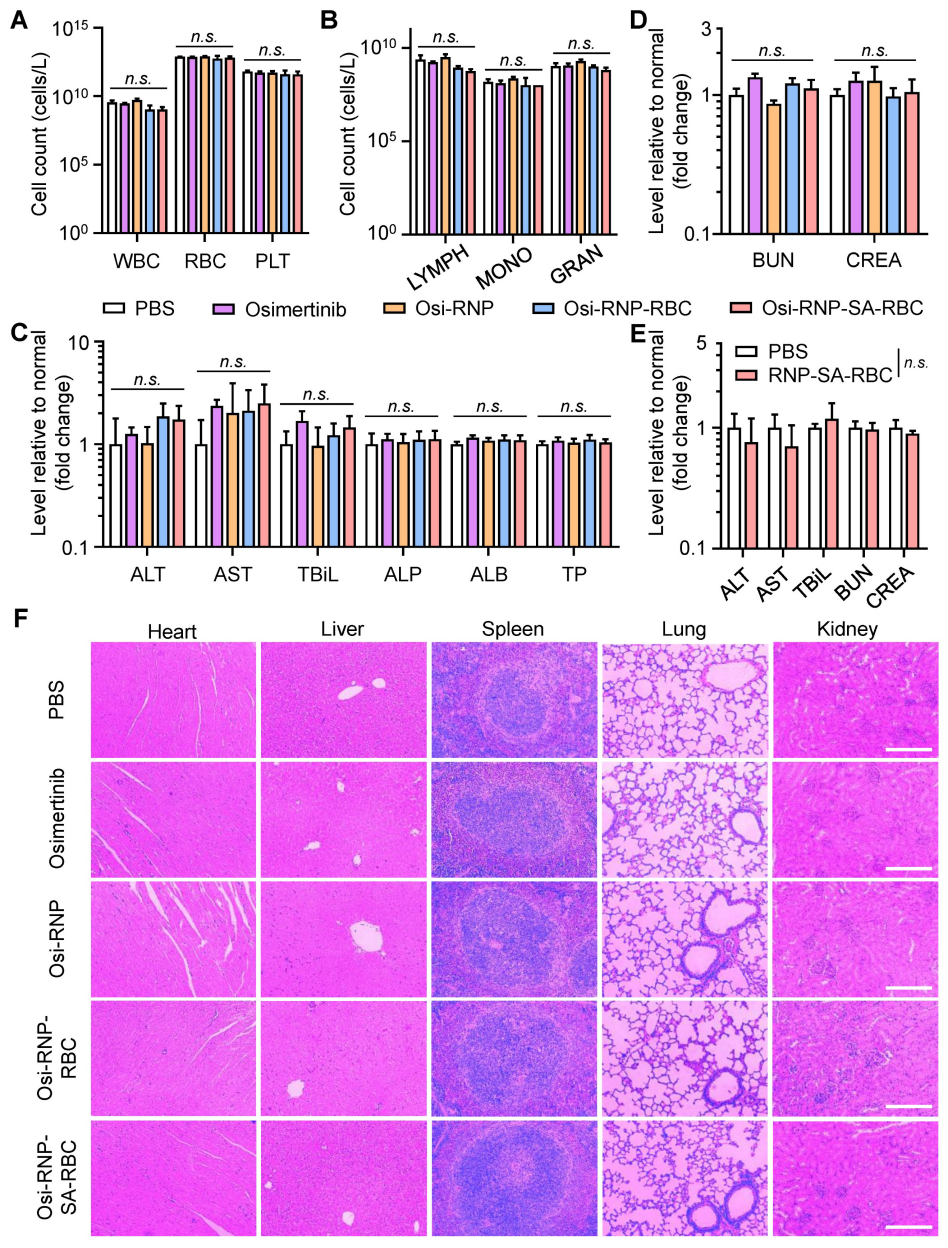
**Biosafety studies of osimertinib-loaded RNP-SA-RBC *in vivo*.** (**A-B**) Amount of complete blood cells (**A**) and key immune cells (**B**) counts in the tumor-bearing mice treated with different groups. WBC, white blood cells; RBC, red blood cells, PLT, platelets; LYMPH, lymphocytes; MONO, monocytes; GRAN, granulocytes. (**C-D**) Comprehensive blood chemistry analysis for liver (**C**) and kidneys (**D**) in various groups. ALT, alanine transaminase; AST, aspartate transaminase; ALP, alkaline phosphatase; TBiL, total bilirubin; ALB, albumin; TP, total protein; BUN, blood urea nitrogen; CRE, creatinine. (**E**) Analysis of key hepatic and renal biomarkers at day 7 following *i.v.* injection of PBS or RNP-SA-RBCs in healthy mice. (**F**) Representative H&E staining of main organs (heart, liver, spleen, lung, and kidneys) in tumor-bearing mice. Scale bar: 200 μm.

Finally, we performed the histological and blood analysis to assess the systemic safety of these therapeutic agents in tumor-bearing mice during the treatment period. To evaluate potential hematological toxicity, blood samples in different groups were collected from the mice at the end-point on day 28. These samples were subjected to analyze the complete blood counts, including total white blood cells (WBC), RBCs, and platelets (PLT). As shown in **Figure 7A**, no significant changes were observed in any of these parameters relative to PBS-treated tumor-bearing mice. Some crucial immune cells in the blood, such as lymphocytes (LYMPH), monocytes (MONO), granulocytes (GRAN), also showed negligible changes in the mice treated with osimertinib, Osi-RNP, Osi-RNP-RBC, and Osi-RNP-SA-RBC when compared to the PBS group (**Figure 7B**). Considering the possibility of these nanomedicines to cause drug-induced injury *in vivo*, a comprehensive blood chemistry analysis was also conducted to evaluate potential systemic toxicity, particularly in the liver and kidneys, two crucial organs in the body responsible for drug clearance. As shown in **Figure 7C**, the levels of some key hepatic biomarkers, including the alanine transaminase (ALT), aspartate transaminase (AST), total bilirubin (TBiL), alkaline phosphatase (ALP), albumin (ALB) and total protein (TP) had no significant variations in the blood, suggesting the limited hepatotoxicity associated with of these nanomedicines. In addition, the biomarkers for kidney functions, including creatinine (CRE) and blood urea nitrogen (BUN), along with key components of the basic metabolic panel, such as the levels of cholesterol (CHO), glucose (GLU), total protein (TP), sodium (Na^+^), and potassium (K^+^), showed no statistically significant differences in the Osi-RNP-SA-RBC-treated mice when compared to those in the PBS-treated control group (**Figure 7D** and **Figure S23**). Conversely, daily oral administration of osimertinib at high dose (25 mg/kg) resulted in observable systemic toxicity (**Figure S24**). Assessments were further conducted in healthy mice to evaluate the biosafety of RNP-SA-RBC as a multifunctional drug carrier *in vivo*. As shown in **Figure 7E** and **Figure S25**, some key hepatic and renal biomarkers in the blood tests, such as the ALT, AST, TBiL, BUN, and CRE, as well as the pathological analysis in healthy mice, presented negligible changes relative to the PBS group. Moreover, spleen sections from the RNP-SA-RBC-treated group exhibited minimal proliferating cell nuclear antigen (PCNA) immunostaining comparable to that of the PBS-treated group, indicating negligible spleen injury of this biomimetic drug delivery platform (**Figure S26**). Additionally, the histological examination of major organs, including the heart, liver, spleen, lungs, and kidneys, from tumor-bearing mice treated with all formulations revealed no significant abnormalities compared to the PBS group (**Figure 7F**). Collectively, these results demonstrated the excellent biosafety of biomimetic drug delivery nanoplatform loading on RBCs for NSCLC treatment* in vivo*.

## Discussion

Lung cancer remains one of the most frequently diagnosed malignancies and the leading cause of cancer-related mortality globally 1. NSCLC is the most prevalent subtype and is characterized by poor clinical outcomes and limited survival rates. For NSCLC patients harboring activating mutations in the EGFR gene, EGFR-TKIs represent a cornerstone of targeted therapy 59. However, despite the initial benefits, the therapeutic window of these agents is narrow, constrained primarily by dose-limiting systemic toxicities arising from prolonged or high-dose treatment regimens 9. Therefore, a major clinical challenge is to optimize the therapeutic window by mitigating dose-dependent resistance and toxicity without compromising antitumor efficacy. While nanomedicine offers a promising strategy to enhance drug delivery efficiency and reduce off-target effects compared to conventional therapies, its application is often limited by insufficient systemic circulation time 60-67.

In this study, we developed a biomimetic drug delivery system for NSCLC treatment by encapsulating osimertinib into biotinylated RBC membrane-camouflaged NPs (RNPs), which were subsequently conjugated onto biotinylated RBCs *via* biotin-SA linkage to generate the RNP-SA-RBC platform. This conjugation yielded a more stable and biocompatible attachment on RBCs surface than direct adsorption (RNP-RBC). Direct NP adsorption onto RBC membranes, particularly at higher loading densities, can induce detrimental effects such as PS exposure and increased membrane rigidity, potentially leading to hemolysis and agglutination 68. In contrast, the biotin-SA conjugation strategy minimizes direct membrane contact, thereby better preserving native RBC structure and function. In addition, pharmacokinetic and biodistribution results demonstrated that the RNP-SA-RBC platform achieved extended blood circulation (1.6-fold) and enhanced tumor accumulation (2.2-fold). This stable surface conjugation effectively minimized premature RNP detachment and reduced clearance by the RES, thereby promoting sustained intravascular retention and tumor accumulation. Consequently, the osimertinib-loaded RNP-SA-RBC formulation significantly improved the therapeutic efficacy at a reduced dose and administration frequency in NSCLC mouse models, while maintaining a favorable biosafety profile. Interestingly, Osi-RNP-SA-RBC demonstrated superior antitumor efficacy compared to Osi-RNP-RBC in a subcutaneous tumor model (1.5-fold increase), underscoring the advantage of stable conjugation for reliable nanocarrier loading and enhanced drug delivery performance. However, in the orthotopic lung cancer model, while Osi-RNP-SA-RBC significantly improved antitumor efficacy relative to free Osimertinib (4.2-fold increase), its performance compared to Osi-RNP-RBC (1.1-fold increase) did not reach statistical significance (*p* > 0.05). This observation may be explained by pulmonary retention of drug-loaded NPs that partially desorb from RNP-RBCs, which could compensate for their lower circulatory stability and yield a therapeutic efficacy comparable to that of the conjugated platform. Collectively, these findings demonstrate that stable RBC conjugation offers significant therapeutic advantages. This platform may hold even particular potential for treating malignancies beyond lung cancer, representing a promising direction for further investigation.

Beyond dose-limiting toxicity, acquired resistance to osimertinib has emerged as a major clinical challenge, including for patients who initially respond to therapy. Currently, therapeutic options after osimertinib resistance are limited, often restricted to systemic chemotherapy or local ablative therapies for selected cases. The evolution of this resistance is complex, driven by diverse mechanisms such as secondary EGFR mutations, bypass pathway activation, phenotypic transformation, oncogenic gene fusions, and cell-cycle gene aberrations 54. While the present study primarily focused on improving the pharmacokinetic profile and therapeutic index of osimertinib, it remains to be determined whether the RBC-conjugated delivery platform could influence resistance-related outcomes. Future work utilizing this platform may yield valuable insights into overcoming or delaying osimertinib resistance, either through optimized monotherapy or rational combination strategies. Apart from TKIs, this versatile biomimetic drug delivery platform demonstrates considerable potential for the systemic administration of diverse antitumor agents 59, 67, 69. By significantly improving the pharmacokinetics and tumor accumulation of these agents, it offers a promising strategy to address multidrug resistance in NSCLC, a condition where insufficient drug delivery and off-target toxicity often compromise therapeutic efficacy 70. Notably, the RBC-based carriers system allows flexible dose modulation and treatment personalization, potentially enabling more tailored therapeutic regimens with an improved efficacy-safety balance. Furthermore, the platform can be adapted to incorporate stimulus-responsive conjugation strategies that exploit distinctive features of the tumor microenvironment, such as acidic pH, elevated redox potential, hypoxia, and overexpressed enzymes 63, 66, 71. These modifications could augment the platform's versatility, specificity, and efficiency, ensuring precise drug delivery to targeted sites. Moreover, the established conjugation methodology could be extended to anchor drug-loaded NPs onto other circulatory blood cells, including macrophages, neutrophils, platelets, and lymphocytes, thereby leveraging their intrinsic inflammatory homing capacities. This approach would facilitate cell-mediated drug delivery systems designed to enhance targeted transport of therapeutics 60-62, 64, 65, 72.

For successful clinical translation, several key limitations of RBC-based delivery systems must also be addressed. Preclinical studies typically employ whole murine or bovine blood, whereas clinical application would necessitate autologous patient blood or donor blood from banks to enable personalized therapy. The choice of RBC source and storage parameters requires careful optimization, as storage-induced mechanical and biochemical alterations can compromise RBC functionality, thereby affecting therapeutic performance and long-term safety 73. Moreover, the complex fabrication process poses significant manufacturing and scalability challenges, with standardized protocols for large-scale production yet to be fully established 74. Consequently, even minor batch-to-batch variations may lead to pharmacological inconsistencies, undermining the system's precision and reliability 52. Post-production storage stability is another critical factor, directly impacting the functional preservation of RBC-based constructs. Additionally, effective sterilization protocols must be optimized across all production stages to ensure safety and efficacy without impairing the therapeutic function 75. Notably, as a biotechnological product, RBC-based delivery systems face an evolving regulatory landscape with specific approval guidelines still in development. Although *i.v.* administration differs from the current clinical route for osimertinib, this work provides important mechanistic and pharmacokinetic insights supporting the translational potential of RBC-based delivery systems for dose reduction, toxicity mitigation, and personalized treatment strategies. With further optimization and extensive ongoing research, these challenges are expected to be progressively resolved, advancing RBC-based therapeutics toward clinical reality.

## Conclusion

In summary, we developed an RBC-conjugated biomimetic nanoplatform for osimertinib delivery to overcome key limitations of conventional EGFR-TKI therapy, including suboptimal circulation time, limited tumor accumulation, and dose-limiting toxicity. By utilizing high-affinity biotin-SA conjugation, the Osi-RNP-SA-RBC construct ensured stable NPs attachment on RBC surfaces, thereby improving tumor accumulation and significantly enhancing drug retention *in vivo*, which consequently resulted in robust antitumor efficacy in both subcutaneous and orthotopic lung cancer models. Notably, these therapeutic benefits were achieved at a reduced osimertinib dose (1.5 mg/kg) and lower weekly administration frequency, while maintaining a favorable safety profile relative to daily high-dose regimens (25 mg/kg). Together, these results highlight the versatile and translational potential of RBC-conjugated biomimetic nanomedicines to enhance the therapeutic index of anticancer agents in NSCLC and potentially other malignancies.

## Materials and Methods

### Isolation of RBCs and membrane derivation

Balb/c mice (8-week-old, female) were anesthetized by intraperitoneal injection of avertin (400 mg/kg). The whole blood was collected *via* cardiac puncture using a syringe pre-rinsed with 100 U/mL heparin solution and transferred into tubes containing 10 U heparin/mL of blood, followed by centrifugation (1000 × g, 10 min, 4 °C) to remove the serum and buffy coat layers. The isolated RBCs pellet was washed twice with cold 1× PBS (pH 7.4) by centrifugation (500 × g, 15 min, 4 °C). The washed RBCs were resuspended in 1× PBS (2.6 × 10^6^ cells/µL) and stored at 4 °C for further studies. To collect the RBC membrane, the washed RBCs were subjected to hypotonic lysis by mixing with a 10-fold volume of cold 0.5× PBS (pH 8 adjusted by 0.1 M NaOH) containing 0.1 mM ethylenediaminetetraacetic acid (EDTA) at 4 °C overnight. The lysed RBCs were then pelleted by centrifugation at 12,000 × g for 10 min at 4 °C. The hemoglobin in the supernatant was discarded, and the pellet was resuspended in a lower ionic strength lysis buffer (0.25× PBS, 0.1 mM EDTA, pH 8.0) and incubated at 4 °C overnight to promote further hemolysis. After centrifugation, the pellets were further suspended in the lowest ionic strength lysis buffer (0.125× PBS, 0.1 mM EDTA, pH 8.0) at 4 °C overnight in order to remove the spectrin-based skeleton. Repeated lysis processes and supplementary washes were carried out until the pellets became pink. The RBC membrane ghosts were finally suspended in 0.2 mM EDTA and stored at -80 °C for further use.

### Preparation and characterization of biotin-RNP

The RNP was first prepared using a classical nanoprecipitation method. Briefly, 1 mL of acid-terminated poly(lactide-co-glycolide) (PLGA, 50:50, 38-54 kDa, Macklin, Shanghai, China) dissolved in acetone (10 mg/mL) was added rapidly into 2 mL of ultrapure water. The solution was placed under a vacuum aspirator until the acetone had completely evaporated. The RBC membrane was then mixed with PLGA cores at the ratio of 1:2 (w/w), following 2 min sonication for 3 cycles to prepare the RNP. To modify the biotin group on RNP surface, 1,2-distearoyl-sn-glycero-3-phosphoethanolamine-N-[biotinyl(polyethylene glycol)-2000)] (DSPE-PEG_2000_-Biotin, AVT Pharmaceutical Tech., China) was dissolved in a mixture of chloroform:methanol (4:1, v/v). The organic solvents were removed in a round-bottom flask using a RV10 rotary evaporator (IKA, Germany) at 40 °C under reduced pressure until a thin film was obtained. The lipid film was subsequently hydrated with RBC membrane by sonication for 2 min (100 mW, 42 kHz, Scientz, China) to facilitate the post-insertion of DSPE-PEG_2000_-Biotin into RBC membrane vesicles with a final total lipid amount of 5 wt% relative to the membrane proteins. The size and zeta potential of NPs in water, 1× PBS and 50% fetal bovine serum (FBS) were determined using dynamic light scattering (DLS, Malvern Zetasizer Nano, UK). Furthermore, sodium dodecyl sulfate-polyacrylamide gel electrophoresis (SDS-PAGE) was performed to evaluate the protein profiles of RNP. Briefly, samples were prepared at a protein concentration of 1 mg/mL in a SDS loading buffer. All samples (32.5 µg) were heated at 90 °C in water bath for 5 min, and subsequently loaded onto a 10% polyacrylamide gel. Electrophoresis was conducted at 120 V for 2 h, and the resulting gel was stained with Coomassie blue to determine protein migration. To visualize the morphology, nanoparticle (1 mg/mL) was deposited onto a carbon-coated copper grid, stained with 2% uranyl acetate for 60 s, and imaged with transmission electron microscope (TEM, 120 kV, Talos L120C G2, Thermo Fisher Scientific).

### Preparation and characterization of RNP-loaded RBC

The biotin-RBCs were first prepared by incubating the RBCs with biotin N-hydroxysuccinimide ester (biotin-NHS, 100 µM) at room temperature for 1 h, followed by washing and centrifugation (500 × g, 5 min, 4 °C) in cold 1× PBS. Streptavidin (SA, 200 µM) was then added in the biotin-RBCs (1.3 × 10^7^ cells/µL) for 30 min with gentle agitation. After removing the excessive SA by centrifugation, the SA-biotin-RBCs were incubated with biotin-RNP at the RBC:RNP numerical ratios of 1:30 to 1:1200 for 1 h in a rotary shaker (12 rpm, Yooning, China). The weight per RNP (W_RNP_) was calculated using the following equation: W_RNP_ = ρ × (4πr^3^)/3, where ρ is the density of PLGA spheres, and r is the radius of the RNP. The estimated numbers of RNPs were calculated by dividing the total weight of PLGA input by the weight per RNP. After washing twice with 1× PBS to remove unbound NPs by centrifugation (100 × g, 5 min, 4 °C), the RNP-loaded RBCs were resuspended in 1× PBS for further characterization. RBCs incubated with biotin-RNPs through non-specific NPs adsorption on RBC surface were used as a control. To determine the number of NPs on RBC surface, the polymeric cores were first prepared by adding a near-infrared fluorophore, 1,1'-dioctadecyl-3,3,3',3'-tetramethylindotricarbocyanine iodide (DiR, 0.1 wt %, excitation/emission = 750/780 nm). The DiR-labelled biotin-RNPs was incubated with SA-biotin-RBCs at different RBC:RNP numerical ratios. After centrifugation at 100 × g for 5 min, the RNP-loaded RBCs (25 µL, 2.6 × 10^6^ cells/µL) were lysed with 125 µL of deionized water under sonication and quantified using a fluorescence microplate reader (Tecan) based on a standard curve of RNP at known concentrations.

In order to evaluate the biosafety of RNP on RBC surface, we performed the assays of hemolysis, agglutination and exposure of phosphatidylserine (PS) as described previously 68. In brief, 50 µL of RNP-RBC and RNP-SA-RBC (2.6 × 10^6^ cells/µL) were suspended in 450 µL of 1× PBS and incubated at 37 °C for 1 h, followed by centrifugation at 500 × g for 5 min at 4 °C. The supernatant was collected to assess the degree of hemolysis by measuring the UV absorbance at 416 nm using a microplate reader. RBCs suspended in the ultrapure water with 100% hemolysis was used as a positive control. For agglutination assay, 50 µL of RNP-RBC and RNP-SA-RBC (2.6 × 10^5^ cells/µL) were added into a round-bottom 96-well plate and incubated at 37 °C for 1 h. Agglutination is visually assessed by the formation of a pellet without a clear boundary. Biotin-RBCs treated with phytohemagglutinin (PHA, 100 µg/mL) to induce RBCs clumping were used as a positive control. To determine the degree of PS exposure, 1 × 10^6^ biotin-RNP-loaded RBCs were incubated with 5 µL of Annexin V-FITC solution (from apoptosis assay kit, Uelandy, China) in 100 µL of binding buffer and incubated for 15 min in the dark. Then, 400 µL of binding buffer was added, and the percentage of PS-exposed RBCs were analysed using a flow cytometer (CytoFLEX, Beckman, US). To study the time-dependent stability of RNP on RBC surface, 50 µL of RNP-RBC or RNP-SA-RBC (RBC:RNP numerical ratio at 1:600) were suspended in 450 µL of 1× PBS containing 5 mM glucose and incubated for 0, 1, 2, 4, 8, and 24 h at 37 °C. After centrifugation (100 × g, 5 min, 4 °C), the RBC pellets were lysed with ultrapure water to evaluate NP release using a fluorescence microplate reader, and the supernatant was collected for the hemolysis assay. To evaluate the conjugation stability of RNP-SA-RBC, an *in vitro* shear stress study was performed by incubating both RNP-RBC and RNP-SA-RBC in an orbital shaker at 220 rpm (corresponding to ~1 dyn/cm²) at 37 °C for 15 min to simulate interstitial fluid shear conditions within the tumor microenvironment. Following incubation, RBCs were collected by centrifugation (500 × g, 5 min, 4 °C), and the associated fluorescence was quantified using a microplate reader. To visualize the RNP conjugation on RBC surface, the cell samples were fixed overnight using 2.5% glutaraldehyde at 4 °C and dehydrated in an increasing ethanol gradient (30% to 100%). Subsequently, the RNP-SA-RBC, RNP-RBC or untreated RBC underwent critical point drying, followed by sputter coating with gold film before imaging with field emission scanning electron microscopy (FE-SEM, SU8020, Hitachi).

### Blood circulation and biodistribution of RBC-loaded RNP* in vivo*

All animal experiments were conducted in accordance with the guidelines of the Institutional Animal Care and Use Committee (IACUC) of Shanghai Jiao Tong University, China. To evaluate the circulation profiles of RBC-loaded RNP *in vivo*, DiR-labeled RNP-SA-RBC, RNP-RBC, and RNP (20 mg/kg, *n* = 3) were intravenously (*i.v.*) injected into the tail vein of Balb/c mice (8-week-old, female). Free DiR dye at the same dosage with DiR-labeled RNP* in vivo* was used as a control. A drop of blood (~30 µL) was collected in heparin-coated tubes from the mice *via* submandibular puncture at 0.05, 0.5, 1, 2, 4, 8, 24, 48, and 72 h post-injection. Then, 20 µL of the collected blood was diluted with 180 µL of 1× PBS and sonicated to obtain a translucent red solution. The fluorescence intensity of DiR-labeled RNP in the blood was measured by microplate reader (excitation/emission = 750/780 nm). To establish a xenograft lung tumor model, 2 × 10^6^ erlotinib-resistant PC9 (PC9/ER) cells in 100 µL PBS were subcutaneously implanted into the right flank of Balb/c nude mice (6-week-old, female). Tumor volumes were measured using a caliper and calculated according to the equation: volume = (length × width^2^)/2. When the tumors volume reached about 50 mm^3^, the mice were *i.v.* administrated with DiR-labeled RNP, RNP-RBC, and RNP-SA-RBC (20 mg/kg, *n* = 3). At 0, 2, 4, 8, 24, 48, and 72 h post-injection, the mice were anesthetized with isoflurane and imaged through In Vivo Imaging System (IVIS, PerkinElmer, US). Additionally, the tumor-bearing mice were euthanized at 8 h after RNP injection, and major tissues (*e.g.,* heart, liver, spleen, lungs, kidneys and tumors) were harvested for IVIS analysis (excitation/emission = 745/800 nm).

### Drug release and pharmacokinetic studies

To prepare the drug-loaded RNP, 1 wt% osimertinib (Osi) was added to the PLGA/acetone solution, following the same procedure described above to fabricate biomimetic RNPs. An additional input of different weight ratios of osimertinib (1, 3, 5, 7, 9 wt%) was introduced into the RNPs. The mixture was then sonicated (2 min, 2 cycles) to facilitate the hydrophobic insertion of osimertinib into the RBC membrane bilayer. After centrifugation (4000 × g, 5 min) using Amicon Centrifugal Filter (100 kDa), the supernatant was collected to measure the drug loading capacity (calculated by dividing the weight of encapsulated drug by the total weight of NPs). The osimertinib concentrations in all samples were determined by high-performance liquid chromatography (HPLC, LC-2050, Shimadzu, Japan) using a C18 analytical column (Agilent, UK). Isocratic elution was performed using 35% of mobile phase A (2 mM ammonium acetate in water) and 65% of mobile phase B (0.1% formic acid in methanol) at a flow rate of 1 mL/min. Known concentration of osimertinib (0-1 µg/mL) were used to generate the standard curve. For drug release study, osimertinib-loaded RNP (~0.9 µg of osimertinib in 500 µL PBS) was loaded into standard regenerated cellulose dialysis bag (molecular weight cut-off: 3500 kDa), clipped and soaked in 10 mL release medium (1× PBS containing 1% Tween 80) of different pH conditions (pH 7.4, pH 6.8 and pH 5.0) at 37 °C with agitation at 100 rpm. Then, 200 µL of the medium was withdrawn and replaced with an equal volume of fresh release medium at designated time points. The osimertinib concentration in the samples was quantified using HPLC to analyse the cumulative drug release curve. For pharmacokinetic study, Balb/c mice were *i.v.* administrated with osimertinib-loaded NPs (RNP, RNP-RBC, RNP-SA-RBC) or free osimertinib (1.5 mg/kg, *n* = 3). At desired time points (0.05, 2, 4, 8, 24, and 48 h), 100 µL of blood samples were collected from submandibular vein and lysed with 100 µL of water in a bath sonicator. The samples were freeze-dried and rehydrated with water (50 µL), and further mixed with acetonitrile (200 µL) to extract osimertinib upon sonication. After centrifugation at 14,000 × g for 10 min, 180 µL of the supernatant from each sample was collected and dried under reduced pressure using a rotary evaporator. The residual solid was reconstituted in 60 µL of acetonitrile and further analysed by HPLC to quantify the osimertinib concentrations in the blood samples.

### Cell viability and apoptosis studies* in vitro*

A human non-small cell lung cancer (NSCLC) cell line (PC9) and erlotinib-resistant NSCLC cell line (PC9/ER, harboring EGFR^E746-A750 del, T790M^) were cultured in RPMI 1640 medium supplemented with 10% FBS and 1% penicillin-streptomycin at 37 °C with 5% CO_2_ under humidified atmosphere. The PC9/ER cells were constantly maintained in the culture medium containing 1 µM erlotinib. To investigate the potential mechanism of RNP uptake by tumor cells, PC9 and PC9/ER cells (2 × 10^5^ cells/well) were seeded in 6-well plate and pretreated for 1 h with specific endocytosis inhibitors, including chlorpromazine (CPZ, 30 μM), methyl-β-cyclodextrin (MβCD, 5 mM), and amiloride hydrochloride (AH, 50 μM), followed by treatment with DiR-labeled RNPs (100 µg/mL) for 4 h. DiR-positive cells were then collected and quantified by flow cytometry, and data were analyzed using FlowJo software. For cell viability analysis, PC9 and PC9/ER cells were first seeded in a 96-well plate (3000 cells/well) and incubated for 24 h. Next, free erlotinib, free osimertinib and/ or osimertinib-loaded RNPs at equivalent drug concentrations (0-10 µM) were added into the cells and incubated for 72 h. Cell viability was evaluated using a fluorometric Resazurin Cell Viability Assay Kit according to manufacturer's protocol (Biotium, US). The relative fluorescence signal of resazurin was quantified using microplate reader (excitation/emission = 560/590 nm). Untreated cells were used as the control group (100% viability). In apoptosis study, 2 × 10^5^ PC9 and PC9/ER cells were seeded in a 6-well plate for 24 h, followed by free erlotinib (1 µM), free osimertinib and/ or osimertinib-loaded RNPs (0.5 µM) treatment for 72 h. Subsequently, the apoptotic cells were stained with Annexin-V-FITC and propidium iodide (PI) following the manufacturer's instruction (Uelandy, China). The cell apoptosis was quantified by flow cytometry and the percentage of apoptotic cells were analysed using FlowJo software.

### Cellular mechanism studies

The PC9/ER cells were first treated with free osimertinib and osimertinib-loaded RNPs at the same drug dosage of 0.5 µM. For the cell cycle analysis, following 72 h of drug treatment, the cells were harvested, washed, and fixed with 70% ice-cold ethanol for 1 h at -20 °C. After washing twice with 1× PBS, the cells were incubated with ribonuclease A (100 μg/mL) and PI solution (50 μg/mL) for 30 min at room temperature prior to analysis by flow cytometry. To evaluate the intracellular ROS and autophagy levels, drug-treated cells were stained with dihydroethidium (DHE, 10 μM, Adamas) for 30 min at room temperature to detect ROS, or monodansylcadaverine (MDC, 50 mM, Yuanye) for 15 min at 37 °C to assess autophagy. After washing twice with 1× PBS, the fluorescence intensity of DHE-positive cells or MDC-positive cells were recorded using a flow cytometer and analysed using FlowJo software. Similarly, DHE- and MDC-positive cells were further examined by fluorescence microscopy. For western blotting analysis, drug-treated cells were lysed in RIPA buffer (50 mM Tris-HCl, 150 mM NaCl, 1% Nonidet P 40, 0.1% SDS, 2 μM EDTA, pH 7.4) supplemented with protease inhibitors and phosphatase inhibitors (Roche), and the protein concentrations were determined using a BCA protein assay kit. Equal amount of proteins (20 µg) was resolved by 10% SDS-PAGE and subjected to gel electrophoresis. The gel was transferred onto a nitrocellulose membrane and stained with primary antibodies (all from Cell Signaling Technology) specific for epidermal growth factor receptor (EGFR) and phospho-EGFR (p-EGFR), extracellular signal-regulated kinase (ERK) and phospho-ERK (p-ERK), protein kinase B (PKB, also known as AKT) and phospho-AKT (p-AKT), and β-actin as a loading control. The membrane was further incubated with horseradish peroxidase (HRP)-conjugated secondary antibodies against mouse/rabbit IgG (Biolegend). The membrane was then washed and incubated with a chemiluminescent HRP substrate (Meilunbio, China) and visualized using a gel imaging analysis system (Tanon-5200 Multi, China).

### *In vivo* antitumor efficacy of Osi-RNP-SA-RBC in subcutaneous lung tumor model

To evaluate the therapeutic efficacy, PC9/ER tumor-bearing Balb/c nude mice (~50 mm^3^) were randomly assigned into 5 groups and *i.v.* administrated with osimertinib-loaded NPs (RNP, RNP-RBC, RNP-SA-RBC) or free osimertinib (1.5 mg/kg, *n* = 4) four times at weekly intervals (at day 0, 7, 14, and 21). Tumor-bearing mice treated with PBS were used as control group. The body weight and tumor volumes of the mice were recorded daily until euthanasia on day 28. The whole blood was collected in an EDTA-coated anticoagulant tube for complete blood counts. The serum was also collected by centrifuging coagulated blood (2,000 × g, 15 min) in an Eppendorf tube for biochemical analysis. Major organs (heart, liver, spleen, lungs and kidneys) and tumors were excised, weighed, and fixed in 4% paraformaldehyde overnight. The fixed organ tissues were then processed for H&E staining, while tumors were additionally subjected to Ki-67, caspase-3, and terminal deoxynucleotidyl transferase dUTP nick end labeling (TUNEL) staining to visualize tumor proliferation and apoptosis. Additionally, to verify the survival potential of this platform, mice were randomly divided into 5 groups (*n* = 5). Mice were monitored daily and euthanized on day 60 or immediately when humane endpoints were reached. These include excessive tumor burden (tumor size > 1000 mm^3^), body weight loss exceeding 15%, tumor ulceration or necrosis, inability to access food and water, or signs of self-mutilation. In the biosafety study, healthy Balb/c mice (8 weeks, female) were *i.v.* injected with either PBS or RNP-SA-RBC (40 mg/kg,* n* = 3). At day 7 after injection, whole blood was acquired for hematological and biochemistry assessments. Moreover, major organs such as the heart, liver, spleen, lungs and kidneys were harvested and fixed in 4% paraformaldehyde for histopathological examination.

### Antitumor effects of Osi-RNP-SA-RBC in an orthotopic lung tumor model *in vivo*

Balb/c nude mice (6-week-old, female) were used to establish an orthotopic lung tumor model *via* lung puncture implantation of a human NSCLC cell line expressing firefly luciferase (A549-Luc), as previously described 76. First, the *in vitro* antitumor against A549-Luc cells was confirmed using cell viability assay. A549-Luc cells were seeded in a 96-well plate (3000 cells/well) and allowed to adhere for 24 h. Cells were then treated with free osimertinib or Osi-RNPs at equivalent drug concentrations (0-20 µM) were added into the cells and incubated for an additional 72 h. Cell viability was subsequently assessed using a fluorometric resazurin assay. For the *in vivo* studies, A549-Luc cell suspension (1 × 10^6^ cells/ 15 µL in PBS) were mixed with an equal volume of Matrigel Matrix (15 µL, BD Biosciences, USA) and kept on ice prior to implantation. Mice were anesthetized with isoflurane and positioned in the right lateral decubitus position. A small transverse incision was made ~0.5 cm below the inferior border of the left scapula to expose the left lung, visible as a pale pink structure under the rib cage. Next, the cell suspension was then carefully injected into lungs, after which the incision was closed with sutures. Tumor development was monitored by bioluminescence imaging following intraperitoneal injection of D-luciferin (150 mg/kg body weight), with images acquired 8 min post-injection. On day 14 after tumor inoculation, mice were randomly grouped (*n* = 3) and treated according to the same regimen used in the subcutaneous lung tumor model. Body weight was recorded every two days, and tumor bioluminescence was monitored every four days until euthanasia on day 28. At the study endpoint, lungs were excised for *ex vivo* bioluminescence imaging, and subsequently fixed in 4% paraformaldehyde for H&E staining.

### Statistical analysis

All data are reported as mean ± standard deviation (*s.d.*) from at least three replicates. All graphical and statistical analyses were performed using GraphPad Prism 8.0 software. Significant differences between different groups were determined using the student's two-tailed t-test (*p*-value: *p < 0.05, **p < 0.01, ***p < 0.001, and *n.s.*: non-significant).

## Supplementary Material

Supplementary figures.

## Abbreviations

AKT: Protein kinase B
ALB: Albumin
ALP: Alkaline phosphatase
ALT: Alanine transaminase
AST: Aspartate transaminase
Biotin-NHS: Biotin N-hydroxysuccinimide ester
BUN: Blood urea nitrogen
Casp3: Caspase-3
CREA: Creatinine
DHE: Dihydroethidium
DiR: 1,1-dioctadecyl-3,3,3,3'-tetramethylindotricarbocyanine iodide
DLS: Dynamic light scattering
EGFR: Epidermal growth factor receptor
ERK: Extracellular signal-regulated kinase
G1: Gap 1 phase
G2: Gap 2 phase
GLU: Glucose
GRAN: Granulocyte
H&E staining: Hematoxylin and eosin staining
HPLC: High-performance liquid chromatography
HPLC: High-performance liquid chromatography
HRP: Horseradish peroxidase
IC_50_: Half-maximal inhibitory concentration
*i.v.*: Intravenous
IVIS: In Vivo Imaging System
K^+^: Potassium
LYMPH: Lymphocyte
M: Mitosis
MDC: Monodansylcadaverine
MAPK: Mitogen-activated protein kinase
MFI: Median fluorescence intensity
MONO: Monocytes
Na^+^: Sodium
NP: Nanoparticle
NSCLC: Non-small-cell lung cancer
PBS: Phosphate-buffered saline
PEG: Polyethylene glycol
PHA: Phytohemagglutinin
PI: Propidium iodide
PI3K: Phosphatidylinositol 3-kinase
PK: Pharmacokinetics
PLGA: Poly(lactic-co-glycolic acid)
PLT: Platelet
PS: Phosphatidylserine
RBC: Red blood cell
RES: Reticuloendothelial system
ROS: Reactive oxygen species
S: Synthesis phase
SA: Streptavidin
SDS-PAGE: Sodium dodecyl sulfate polyacrylamide gel electrophoresis
SEM: Scanning electron microscopy
TBiL: Total bilirubin
TEM: Transmission electron microscope
TKI: Tyrosine kinase inhibitor
TP: Total protein
WBC: White blood cell
