# Supplementary material for: Red blood cell-conjugated biomimetic nanomedicine for enhanced therapy of non-small cell lung cancer
Source: Theranostics. 2026 Jan 21;16(8):4147–68. doi: 10.7150/thno.121646 (PMC12905782; doi:10.7150/thno.121646)
Supplement: Supplementary file 1 — Supplementary figures. [file thnov16p4147s1.pdf]

Supporting Information

Red blood cell-conjugated biomimetic nanomedicine for enhanced therapy of non-small cell lung cancer

Seok Theng Chiang[1,2,#], Yueping Jin[3,#], Qian Zhao[4], Hongju Ling[5], Qinghua Xia[5], Tianzhen Han[2], Rongxiu Li[2], Weidong Li[4], Zhaohui Lan[4], Xiangzhao Ai[2*], Haijiao Lu[1*]*

[1] Department of Respiratory and Critical Care Medicine, Shanghai Chest Hospital, Shanghai Key Laboratory of Thoracic Tumor Biotherapy, Shanghai Jiao Tong University, School of Medicine, Shanghai, 200030, China. E-mail: H. Lu (luhaijiao@shsmu.edu.cn)

[2] Department of Bioengineering, School of Life Sciences and Biotechnology, Shanghai Jiao Tong University, Shanghai 200240, China. E-mail: X. Ai (xzai@sjtu.edu.cn)

[3] Shanghai Lung Cancer Center, Shanghai Chest Hospital, Shanghai Jiao Tong University, School of Medicine, Shanghai 200030, China.

[4] Center for Brain Health and Brain Technology, Global Institute of Future Technology, Shanghai Jiao Tong University, Shanghai 200240, China. E-mail: Z. Lan (zhaohuilan@sjtu.edu.cn)

[5] Urology department, Shandong Provincial Hospital, Jinan, 250021, China

Supplementary Figures

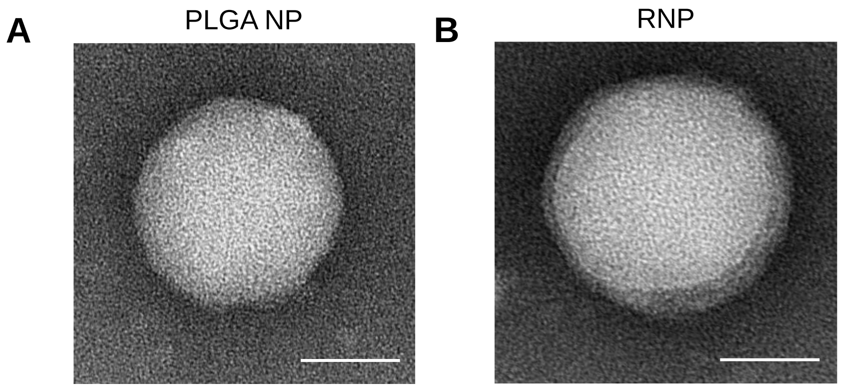

Figure S1. TEM imaging of (**A**) PLGA NP (core) and (**B**) RNP (core-shell). Scale bar: 40 nm.

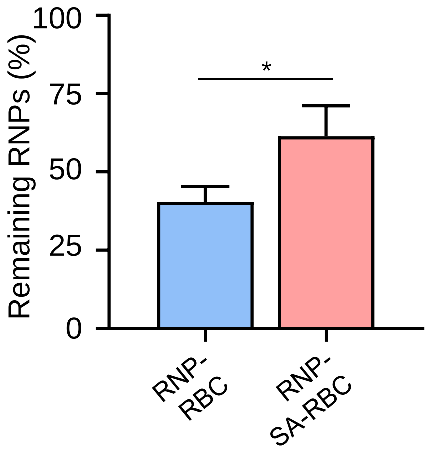

Figure S2. Percentage of RNPs detached from RBCs and SA-RBCs under interstitial shear conditions, simulated using an orbital shaker system at 220 rpm (representing ~1 dyn/cm^2) for 15 min at 37 °C.

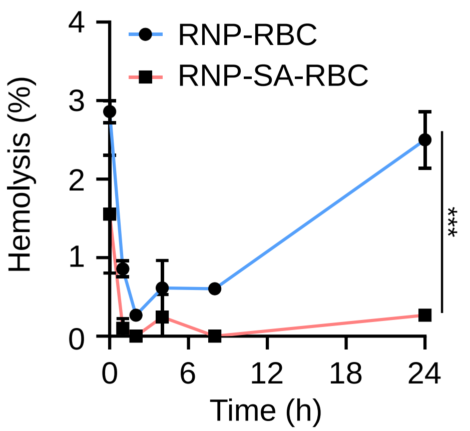

Figure S3. The hemolysis level of RBCs after incubation with biotin-RNPs in a time-dependent manner over 24 h.

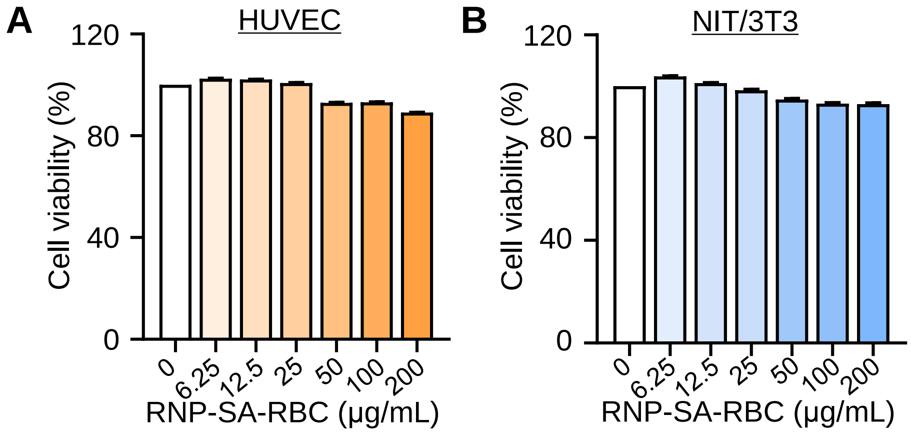

Figure S4. Cell viability analysis of HUVEC and NIT/3T3 cells upon RNP-SA-RBC treatment at different concentrations.

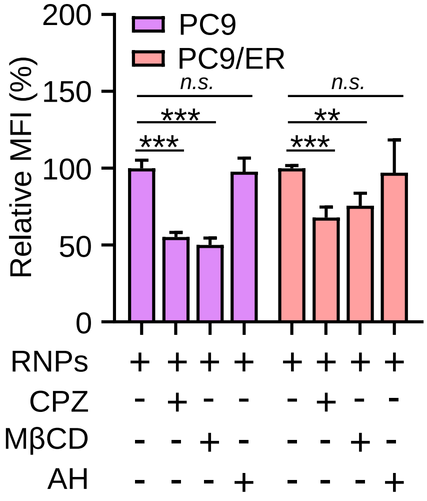

Figure S5. MFI of DiR-labeled RNP uptake by both PC9 and PC9/ER cells pre-treated with different endocytic inhibitors, such as methyl-β-cyclodextrin (MβCD, 5 mM), chlorpromazine (CPZ, 30 μM), and amiloride hydrochloride (AH, 50 μM), for 1 h prior to 4 h incubation with RNP. MβCD, CPZ, and AH inhibit caveolae-mediated endocytosis, clathrin-mediated endocytosis, and macropinocytosis, respectively.

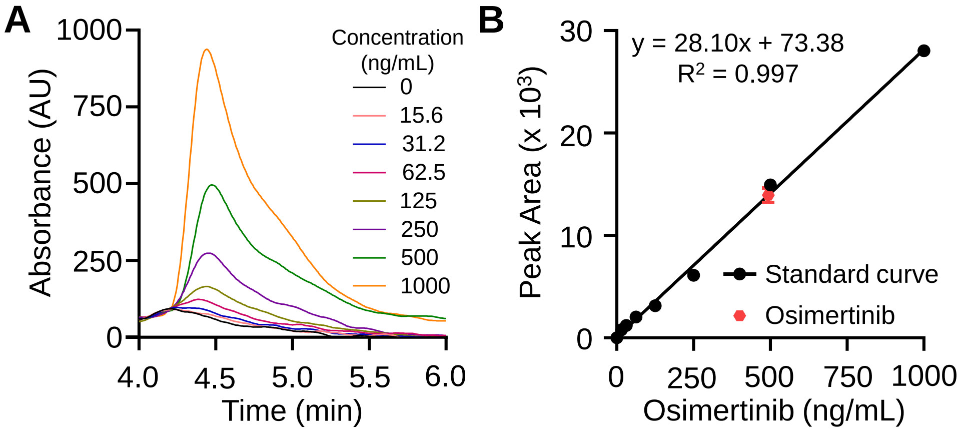

Figure S6. (**A**) Representative HPLC chromatogram and (**B**) standard curve correlating HPLC peak areas with osimertinib concentrations ranging from 0 to 1 μg/mL.

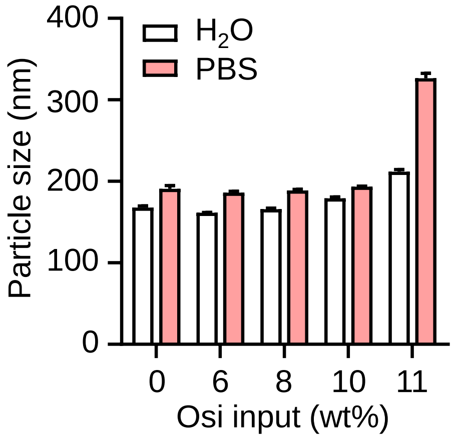

Figure S7. The particle size of RNPs after loading with different percentages of osimertinib input.

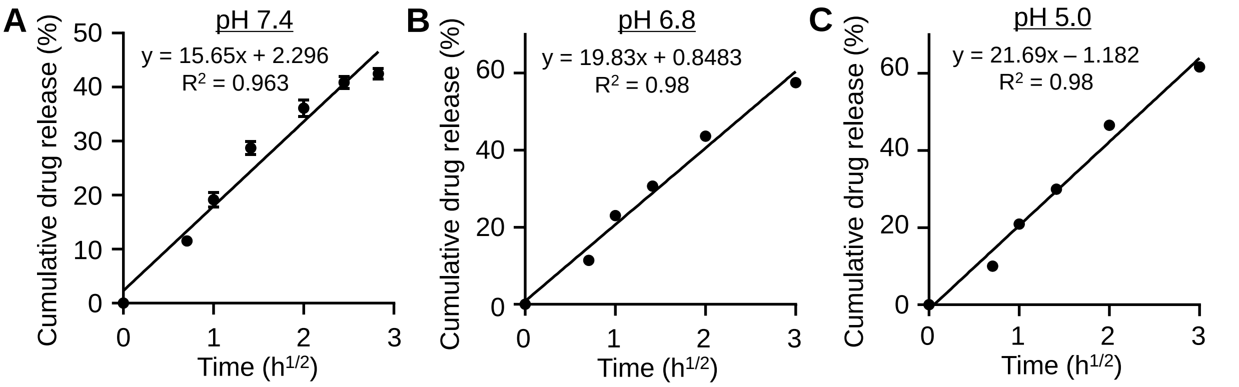

Figure S8. Cumulative drug release kinetics of Osi-RNPs under (**A**) pH 7.4, (**B**) pH 6.8 and (**C**) pH 5.0 using the Higuchi model.

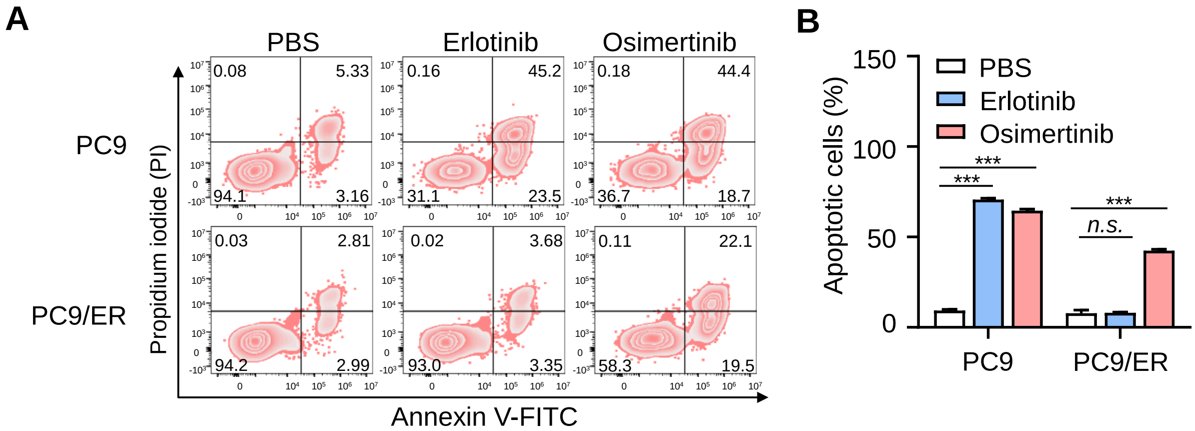

Figure S9. (**A**) Representative flow cytometry plots and (**B**) quantification of apoptotic cells in PC9 and PC9/ER cells following Annexin V-FITC/PI staining upon free erlotinib (1 μM) and osimertinib (0.5 μM) treatment for 72 h.

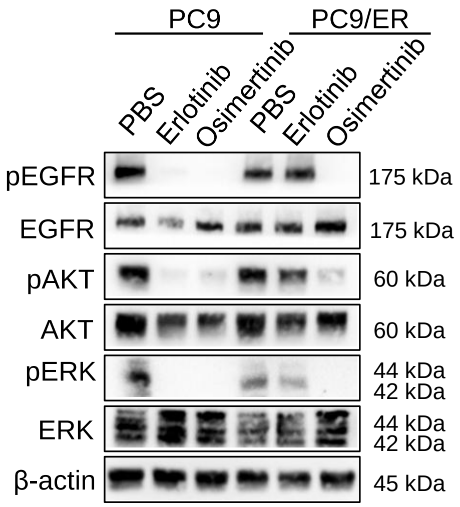

Figure S10. Western blot analysis of EGFR signaling in the PC9 and PC9/ER cells exposed to erlotinib and osimertinib for 72 h.

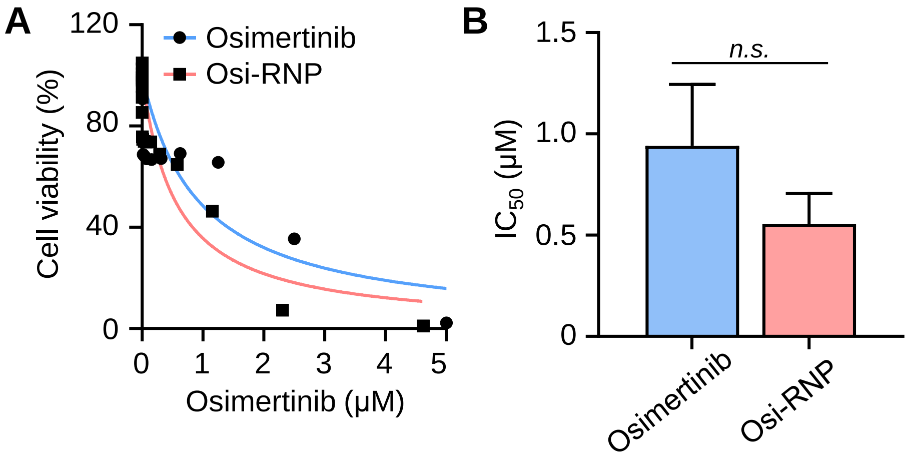

Figure S11. (**A**) Cell viability and (**B**) IC_{50} values of PC9/ER cells after 72 h treatment with free osimertinib and Osi-RNPs at the same concentrations.

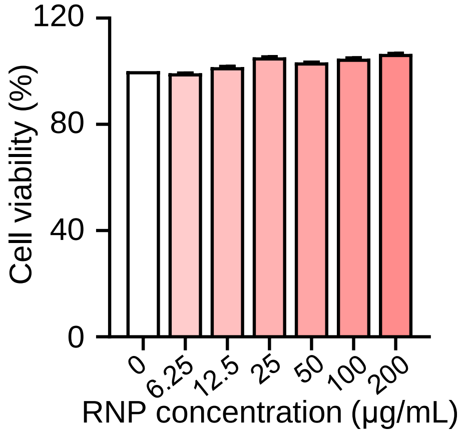

Figure S12. Cell viability analysis of PC9/ER cells upon RNPs treatment at different concentrations.

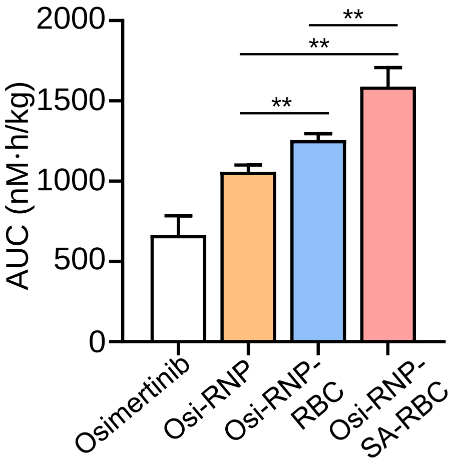

Figure S13. The area under the plasma concentration-time curve (AUC) of osimertinib in serum after *i.v.* administration of free osimertinib, Osi-RNPs, Osi-RNP-RBCs and Osi-RNP-SA-RBCs (1.5 mg/kg, $n = 3$).

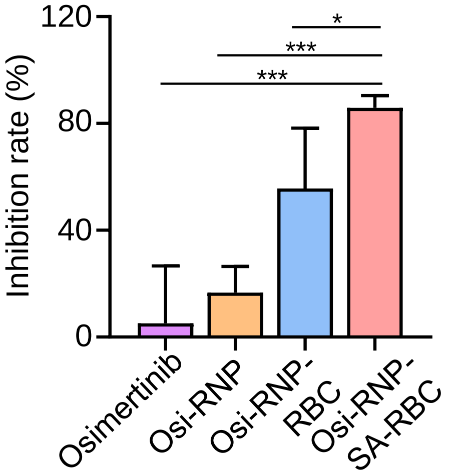

Figure S14. The tumor growth inhibition rates of the different treatment groups in comparison to the PBS group at the experimental endpoint (day 28).

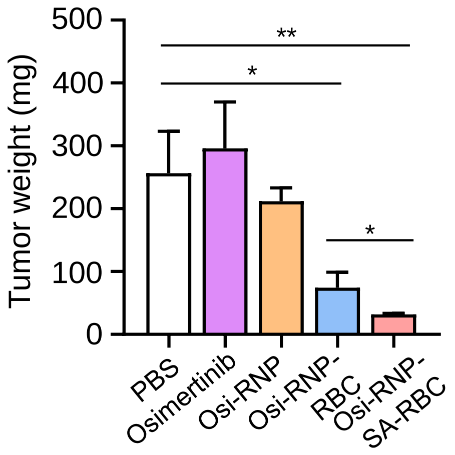

Figure S15. Excised tumor weights from mice collected at the endpoint of the therapeutic regimen (day 28).

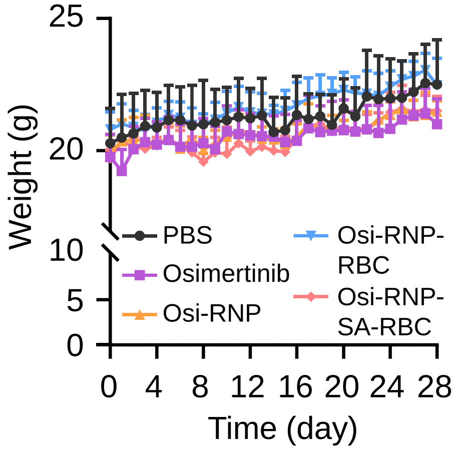

Figure S16. Changes in body weight of subcutaneous PC9/ER tumor-bearing mice during the therapeutic process.

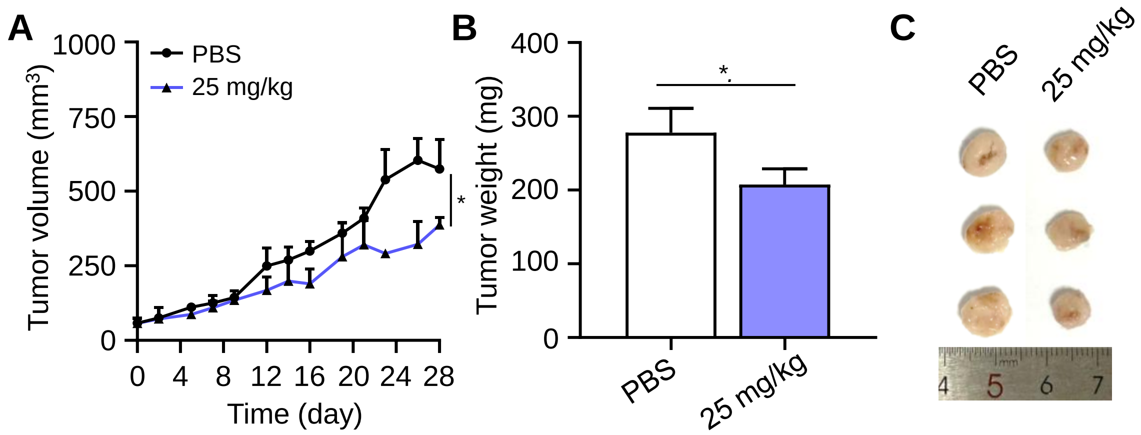

Figure S17. Antitumor study of osimertinib at 25 mg/kg administrated orally in drug-resistant tumor model in vivo. (**A**) Average tumor growth curves of tumor-bearing mice after treated with PBS and 25 mg/kg osimertinib (once a week for four doses). (**B**) Excised tumor weights from mice collected at the endpoint of the therapeutic regimen (day 28). (**C**) Tumor images collected from mice at the endpoint of different therapeutic regimens.

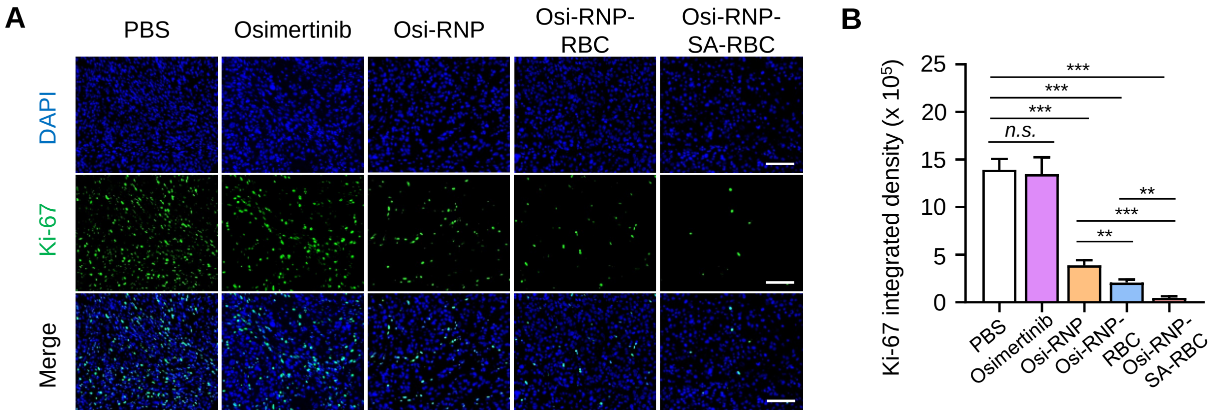

Figure S18. (**A**) Representative images of Ki-67 cell proliferation assay of tumors from various groups. Scale bar: 100 µm. (**B**) Quantitative fluorescence analysis of Ki67-expressing cells in PC9/ER tumor sections.

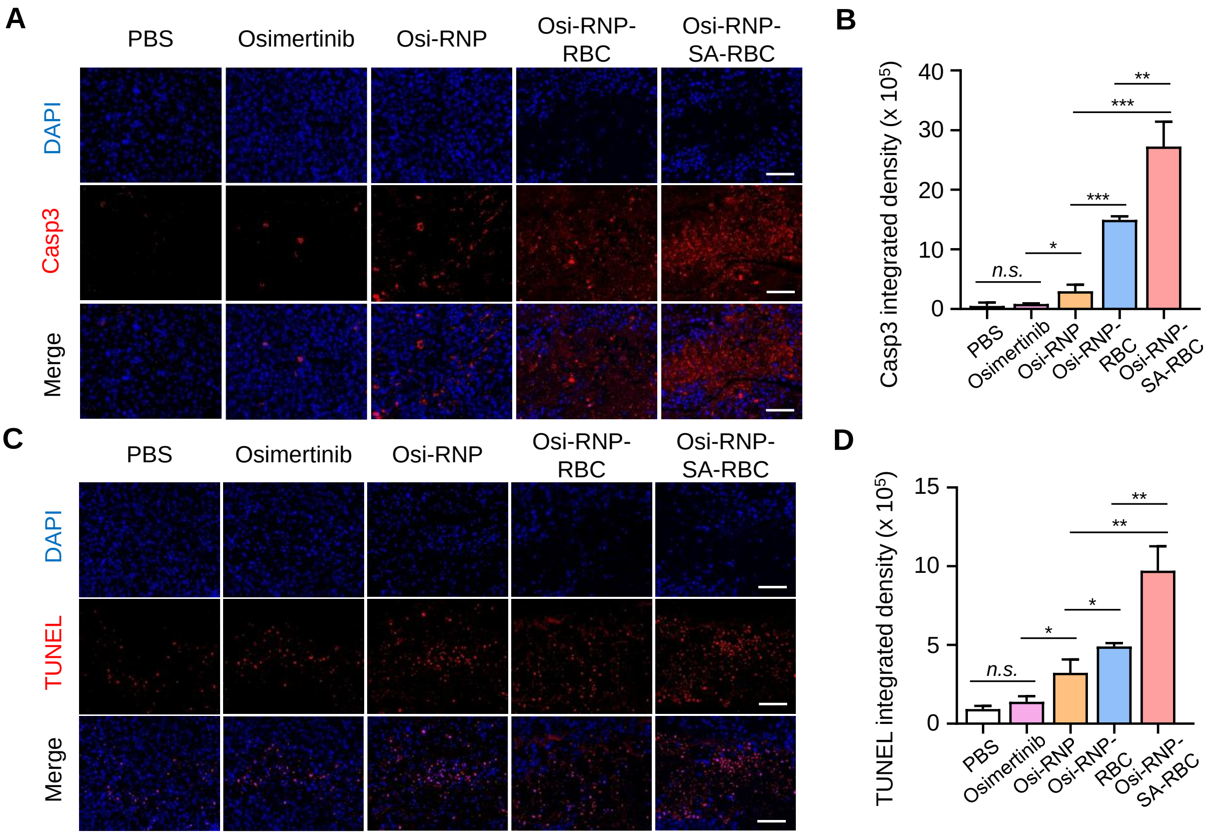

Figure S19. (**A**) Representative tumor section images and (**B**) quantitative analysis from caspase-3 immunohistochemical staining. (**C**) Representative images of tumor sections and (**D**) quantitative fluorescence measurements obtained from the TUNEL assay.

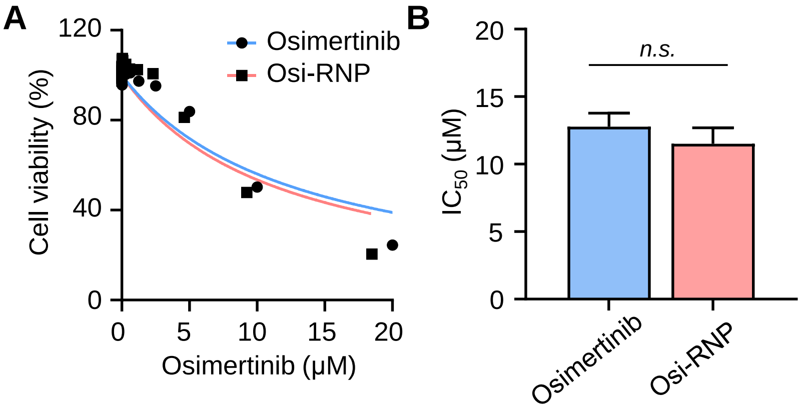

Figure S20. (**A**) Cell viability and (**B**) IC_{50} values of A549 cells after 72 h treatment with free osimertinib and Osi-RNPs at the same concentrations.

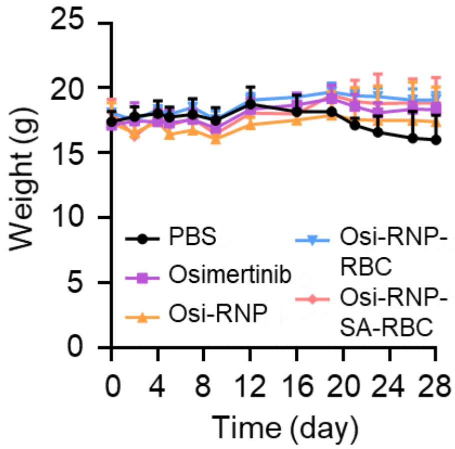

Figure S21. Changes in body weight of orthotopic A549-Luc lung tumor–bearing mice throughout the therapeutic regimen.

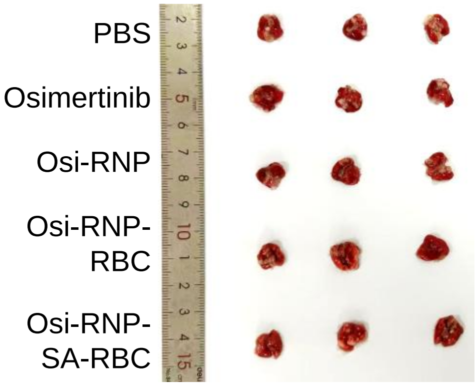

Figure S22. Representative images of excised lungs collected from orthotopic lung tumor–bearing mice at the study endpoint following different therapeutic regimens.

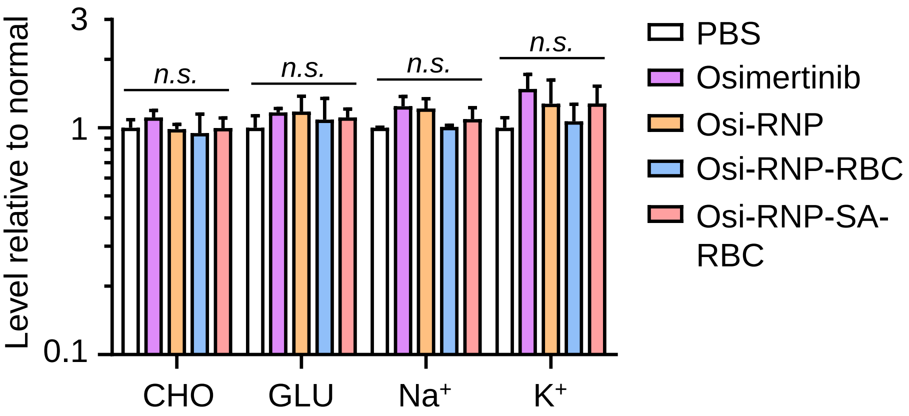

Figure S23. Additional metabolic panel from the comprehensive blood chemistry analysis. CHO, cholesterol; GLU, glucose; TP, total protein; Na^+, sodium; K^+, potassium.

To demonstrate the safety advantage of our approach, we included a high-dose oral control (25 mg/kg), approximating the clinically approved 80 mg/day dose in NSCLC patients. Compared with PBS, mice receiving high-dose oral osimertinib exhibited evident toxicity, including periorbital edema, alopecia, hematological abnormalities (elevated white blood cells, platelets, and granulocytes), and altered serum biochemistry (increased AST, ALP, and CREA), indicating systemic toxicity. Histopathological analysis revealed mild hepatocellular damage with swollen, pale cytoplasm, along with thickened alveolar walls and reduced airspace, indicating hepatic and pulmonary inflammation and injury.

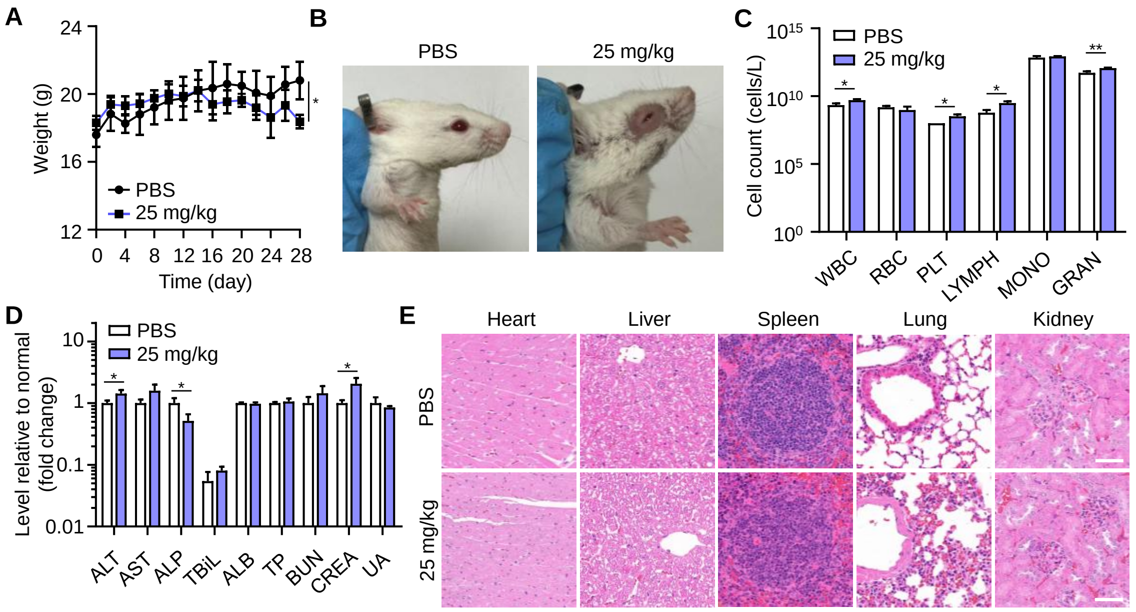

Figure S24. Biosafety studies of 25 mg/kg osimertinib administrated daily in vivo. (**A**) Body weights of mice during the therapeutic process. (**B**) Pictures of mice showing ocular irritation by osimertinib. (**C**) Amount of blood cells in mice treated with PBS and osimertinib. WBC, white blood cells; RBC, red blood cells, PLT, platelets; LYMPH, lymphocytes; MONO, monocytes; GRAN, granulocytes. (**D**) Comprehensive blood chemistry analysis for liver and kidneys. ALT, alanine transaminase; AST, aspartate transaminase; ALP, alkaline phosphatase; TBiL, total bilirubin; ALB, albumin; TP, total

protein; BUN, blood urea nitrogen; CRE, creatinine; UA, uric acid. (**E**) Representative H&E staining of main organs (heart, liver, spleen, lung, and kidneys) in mice. Scale bar: 50 μm.

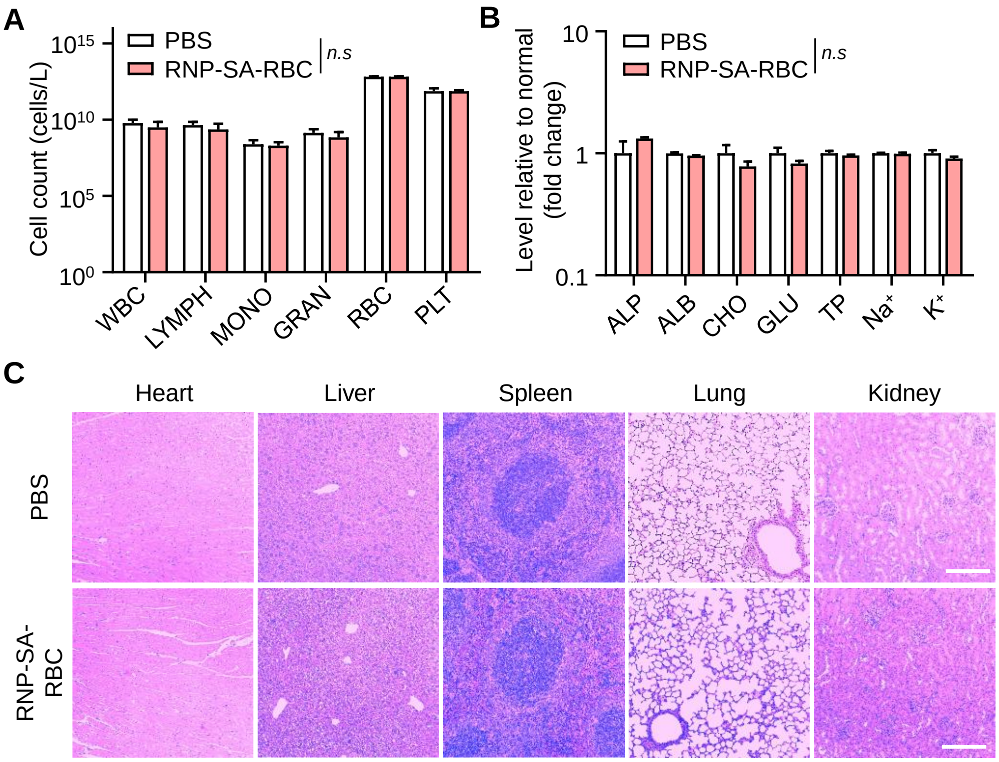

Figure S25. (**A**) Analysis of blood cells amounts at day 7 post-injection with PBS and RNP-SA-RBCs. WBC, white blood cells; LYMPH, lymphocytes; MONO, monocytes; GRAN, granulocytes; RBC, red blood cells; PLT, platelets. (**B**) Comprehensive blood chemistry analysis at day 7 following *i.v.* injection of PBS or RNP-SA-RBCs. ALP, alkaline phosphatase; ALB, albumin; CHO, cholesterol; GLU, glucose; TP, total protein; Na^+, sodium; K^+, potassium. (**C**) H&E staining of main organs (heart, liver, spleen, lung, and kidneys) at day 7 after PBS or RNP-SA-RBC administration. Scale bar: 200 μm.

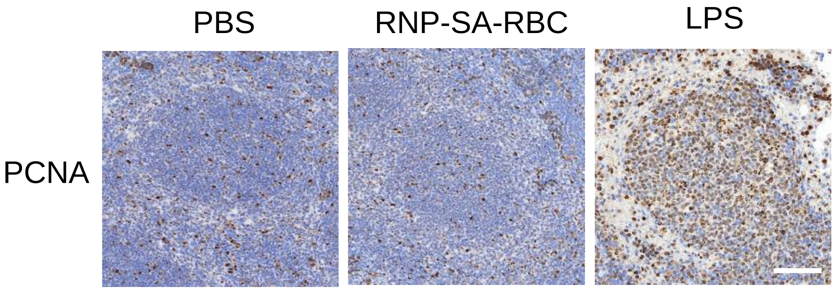

Figure S26. PCNA immunostaining of spleen sections at day 7 post-injection with PBS or RNP-SA-RBC. Mice intraperitoneally injected with 10 mg/kg LPS for 24 h served as the positive control. Scale bar: 100 μm.
